# Long-range supercurrents through a chiral non-collinear antiferromagnet in lateral Josephson junctions

**DOI:** 10.1038/s41563-021-01061-9

**Published:** 2021-08-05

**Authors:** Kun-Rok Jeon, Binoy Krishna Hazra, Kyungjune Cho, Anirban Chakraborty, Jae-Chun Jeon, Hyeon Han, Holger L. Meyerheim, Takis Kontos, Stuart S. P. Parkin

**Affiliations:** 1 Max Planck Institute of Microstructure Physics, Halle (Saale), Germany; 2 Laboratoire de Physique de l’Ecole Normale Supérieure, ENS, Université PSL, CNRS, Sorbonne Université, Université de Paris, Paris, France

**Keywords:** Superconducting properties and materials, Spintronics

## Abstract

The proximity-coupling of a chiral non-collinear antiferromagnet (AFM)^1–5^ with a singlet superconductor allows spin-unpolarized singlet Cooper pairs to be converted into spin-polarized triplet pairs^6–8^, thereby enabling non-dissipative, long-range spin correlations^9–14^. The mechanism of this conversion derives from fictitious magnetic fields that are created by a non-zero Berry phase^15^ in AFMs with non-collinear atomic-scale spin arrangements^1–5^. Here we report long-ranged lateral Josephson supercurrents through an epitaxial thin film of the triangular chiral AFM Mn_3_Ge (refs. ^3–5^). The Josephson supercurrents in this chiral AFM decay by approximately one to two orders of magnitude slower than would be expected for singlet pair correlations^9–14^ and their response to an external magnetic field reflects a clear spatial quantum interference. Given the long-range supercurrents present in both single- and mixed-phase Mn_3_Ge, but absent in a collinear AFM IrMn^16^, our results pave a way for the topological generation of spin-polarized triplet pairs^6–8^ via Berry phase engineering^15^ of the chiral AFMs.

## Abstract (web summary)

Long-range lateral Josephson supercurrents are observed in a chiral non-collinear antiferromagnet, indicating topologically generated triplet pairing states.

## Main

Spin-polarized triplet Cooper pairs^6–8^ can carry a non-dissipative spin angular momentum over a long distance^9–14^ and are a key ingredient for superconducting spintronics. This nascent research field aims to develop new types of device where spin and charge degrees of freedom are controllable by superconducting phase coherence^6–8^. Notably, recent experiments and theories have established that inhomogeneous exchange fields^9,10^ in real-space and/or spin-orbit fields^17–19^ in reciprocal/*k*-space at engineered superconductor/ferromagnet interfaces can proximity-generate spin-polarized triplet pairing states via spin-mixing and spin-rotation processes^6–14^. There remain, however, several outstanding technical issues, in particular, how to simplify the necessary multiple ferromagnet elements, how to create the desired non-collinear alignment of their magnetization directions below the exchange length scales (a few nanometres) and how to avoid stray-field-driven screening supercurrents and Abrikosov vortex nucleation in adjacent superconductors when patterned to submicron lateral dimensions.

To address these issues, we consider here chiral non-collinear antiferromagnets (AFMs)^1–5^ whose total net magnetization is essentially zero and thereby lack stray fields. The main concept is that the chiral non-collinear atomic-scale spin arrangements in real-space and the resulting fictitious magnetic fields (as large as roughly 100 tesla, refs. ^2–5^) from a non-vanishing Berry phase^15^ in *k*-space, effectively fulfil the spin-mixing and spin-rotation mechanisms^6–14^ required for singlet-to-triplet pair conversion.

**Fig. 1:**
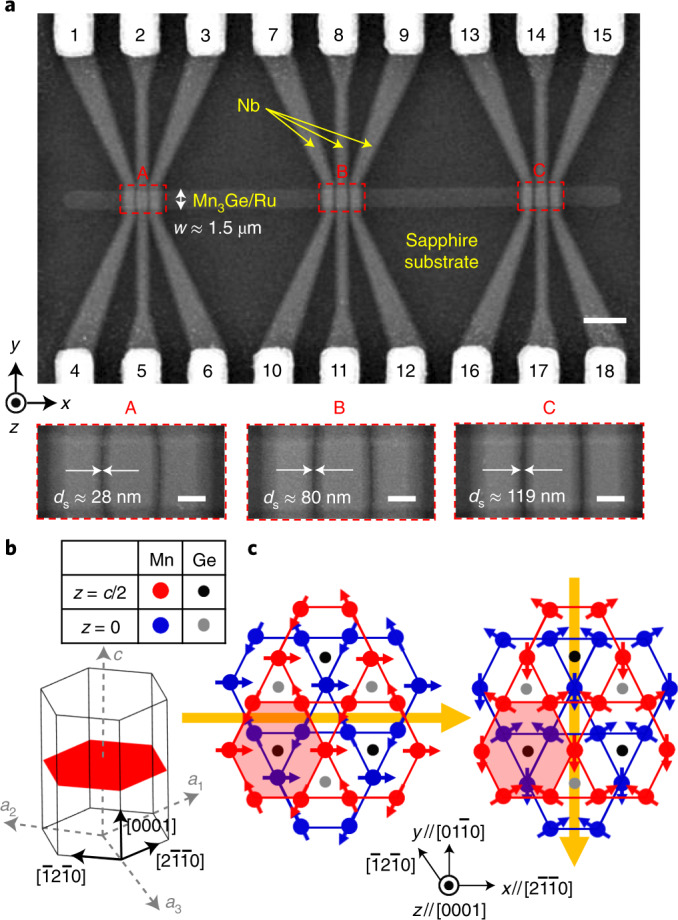
Chiral non-collinear AFM JJs. **a**, Scanning electron micrographs of the fabricated JJs, consisting of a triangular chiral antiferromagnetic Mn_3_Ge (40-nm-thick) spacer and multiple superconducting Nb (50-nm thick) electrodes. These Nb electrodes are laterally edge-to-edge separated by 28–119 nm from each other (bottom). The upper scale bar is 3 µm, lower scale bars are 0.5 µm. Note that the 5-nm-ultrathin Ru underlayer serves as a buffer layer (Methods). **b**,**c**, Crystal structure (**b**) and 120^o^ chiral antiferromagnetic configuration (**c**) of *D*0_19_-Mn_3_Ge. Two layers of Mn and Ge atoms are stacked along the *c* axis (// *z* axis) where red and black circles (blue and grey) represent Mn and Ge atoms lying in the *z* = *c*/2 (*z* = 0) planes, respectively. The probable antiferromagnetic configurations are presented in **c** when an external magnetic field is applied along $[{2\bar 1\bar 10}]$ (left) and $[{0\bar 110}]$ (right). In each layer, Mn atoms form a Kagome-type lattice and their magnetic moments (blue or red arrows) constitute a 120° antiferromagnetic structure. The orange arrows indicate a weak uncompensated magnetization.

We demonstrate this radically different approach by fabricating Josephson junctions (JJs), in which several superconducting Nb electrodes are laterally separated by an epitaxial thin film of the triangular chiral antiferromagnetic Mn_3_Ge (refs. ^4,5^; Fig. 1a, see Methods for device fabrication) and by probing long-ranged Josephson supercurrents^6–14^ that show a clear magnetic field interference. The *D*0_19_-Mn_3_Ge (ref. ^20^) epitaxial film used in this study (see Supplementary Text for structural analysis and magnetic properties) has a hexagonal lattice with magnetic Mn atoms forming Kagome-type sublattices stacked along the *c* axis (// *z* axis // [0001] in Fig. 1b), isostructural with Mn_3_Sn (ref. ^2^). Below the Néel temperature *T*_Néel_ of roughly 380 K (refs. ^3–5,20^), Mn magnetic moments in the *x–y* Kagome plane form triangular spin structures and lead to a non-collinear AFM configuration with a uniform negative vector chirality^3,4^ caused by the Dzyaloshinskii–Moriya interaction. Unlike Mn_3_Sn (ref. ^2^), the chiral AFM phase in Mn_3_Ge is robust to low temperature^3–5^ (*T*), which allows one to investigate the transport properties of Josephson supercurrents associated with Berry curvature in this chiral non-collinear AFM. Crucially, the observed decay length of the Josephson supercurrents through the Mn_3_Ge is far beyond the predicted singlet coherence length $\xi _{\mathrm{{singlet}}}^{\mathrm{{AFM}}}$ (refs. ^9–14^) and such long-ranged supercurrents are absent in JJs with a collinear AFM IrMn (ref. ^15^), providing an experimental indication of topologically generated triplet pairing states.

**Fig. 2:**
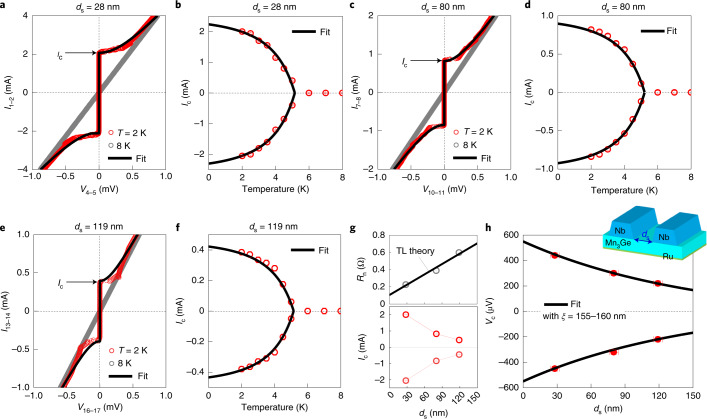
Long-ranged supercurrents through a chiral non-collinear AFM. **a**,**c**,**e**, Zero-field current–voltage *I–V* characteristics of Nb/Mn_3_Ge/Nb JJs with different edge-to-edge separations *d*_s_ = 28 (**a**), 80 (**c**) and 119 nm (**e**), taken above (grey) and below (red) the superconducting transition of the Nb electrodes. The black solid lines are fitting curves to determine the Josephson critical current *I*_c_. **b**,**d**,**f**, Associated temperature *T* dependence of *I*_c_ with *d*_s_ = 28 (**b**), 80 (**d**) and 119 nm (**f**). The black solid lines are theoretical fits to estimate the zero-temperature *I*_c_. **g**, Normal-state zero-bias resistance *R*_n_ (top) and *I*_c_ (bottom) of the JJs versus *d*_s_. From *R*_n_(*d*_s_), we extract the resistance-area product of Nb/Mn_3_Ge interfaces to be 1 mΩ µm^2^ and the effective resistivity for the Mn_3_Ge (40 nm)/Ru (5 nm) track to be 26 µΩ cm, using a standard transmission line (TL) theory (black line, see Supplementary Text for comparison with Hall-bar devices). **h**, Characteristic voltage *V*_c_ = *I*_c_*R*_n_ as a function of *d*_s_, from which the decay length of the Josephson coupling through the Mn_3_Ge spacer is determined using an exponential decay function (black curves).

Figure 2a,c,e shows zero-field current–voltage *I–V* curves of the Nb/Mn_3_Ge/Nb JJs with several edge-to-edge separation distances, *d*_s_ = 28, 80 and 119 nm, across the superconducting transition of the Nb electrodes. All the JJs exhibit clear Josephson *I–V* characteristics that are not strongly hysteretic and which are thus in the overdamped regime, indicating a low resistance-capacitance product^21^. The *T*-dependent Josephson critical current can be approximately described by^22^ $I_{\mathrm{c}}\left( T \right) \approx I_{\mathrm{c}}\left( 0 \right)( {1 - \frac{T}{{T_{\mathrm{c}}}}})^\alpha$ (black lines in Fig. 2b,d,f), where *T*_c_ is the superconducting transition temperature at the Nb/Mn_3_Ge interfaces. Using *α* = 0.50–0.55, we obtain the zero-temperature critical currents |*I*_c_(0)| = 2.27, 0.90 and 0.43 mA for *d*_s_ = 28, 80 and 119 nm, respectively. Note that these values are 1–2 orders of magnitude smaller than the depairing critical current in superconducting microbridges formed from Nb thin films^23^.

With increasing *d*_s_, the normal-state zero-bias resistance *R*_n_ increases linearly whereas *I*_c_ decays strongly (Fig. 2g), as expected from the diminishment of proximity-induced Cooper pairs in a longer Mn_3_Ge spacer. To quantify the decay length *ξ* of the supercurrents, we fit the *d*_s_-dependent characteristic voltage $V_{\mathrm{c}}\left( {d_{\mathrm{s}}} \right) = I_{\mathrm{c}}R_{\mathrm{n}}\left( {d_{\mathrm{s}}} \right)$ at *T* = 2 K (Fig. 2h) using an exponential decay function, ${{\mathrm{exp}}}\left( { - \frac{{d_{\mathrm{s}}}}{\xi }} \right)$^24–26^, where we take the dirty junction regime^21^ in which the mean free path is shorter than any other characteristic lengths. Note that in case of the AFM spacer, the proximity-induced pair correlations decay monotonically without an oscillatory behaviour (0–*π* phase transition, that is, characteristic of ferromagnet spacers) due to the microscopic cancellation of phase shifts through alternating up and down magnetic moments^24–26^. The estimated *ξ* = 155–160 nm is significantly longer than the exchange-field-driven pair breaking and decay of spin-unpolarized singlet supercurrents in the AFM, $\xi _{\mathrm{{singlet}}}^{\mathrm{{AFM}}} \approx \sqrt {\frac{{\hbar D}}{{2E_{\mathrm{{ex}}}}}}$ = 1–3 nm. Here $D = \frac{{\hbar ^2\left( {3\pi ^2} \right)^{2/3}}}{{3m_ne^2n^{1/3}\rho }}$ is the diffusion coefficient, *e* is the electric charge, *m*_*n*_ is the effective electron mass that is assumed to be the free-electron *m*_0_ = 9.1 × 10^−31^ kg, *n* is the electron carrier density (1 × 10^19^ cm^−3^ at *T* = 2 K)^27^ and *ρ* is the resistivity (50–90 µΩ cm at *T* = 2 K)^3–5,27^ of the Mn_3_Ge. *E*_ex_ ≅ $2\pi k_{\mathrm{B}}T_{{{{\rm{N}}{\acute{{\rm{e}}}}{\rm{el}}}}}$ is the AFM exchange energy of the Mn_3_Ge. In contrast, provided that spin-flip scattering and spin-orbit scattering are frozen^12,13^, spin-polarized triplet supercurrents can decay over a much longer length scale^9–14^ that is limited by a thermal coherence length, $\xi _{\mathrm{{triplet}}}^{\mathrm{{AFM}}} \approx \sqrt {\frac{{\hbar D}}{{2\pi k_{\mathrm{B}}T}}}$ = 33–46 nm at *T* = 2 K, which is in reasonable agreement with what we obtain. This long-range nature is one of the strongest indications of proximity-generated triplet pairing states^6–14^.

It is also worth noting that the values of *ξ* = 155–160 nm that we find in our system are two orders of magnitude larger than typical values (few nanometres) of the spin-diffusion length of chiral AFM Mn_3_X (X = Ge (ref. ^28^) or Sn (ref. ^29^)) thin films, quantifying how far out-of-equilibrium spin polarization propagates. This suggests that in such a particular class of antiferromagnetic topological semimetals, the transfer and relaxation mechanisms of equilibrium spin carried by triplet Cooper pairs^6,17,30^ may differ fundamentally from those of non-equilibrium spin by normal unpaired electrons. Further experimental and theoretical studies are required for a detailed understanding.

**Fig. 3:**
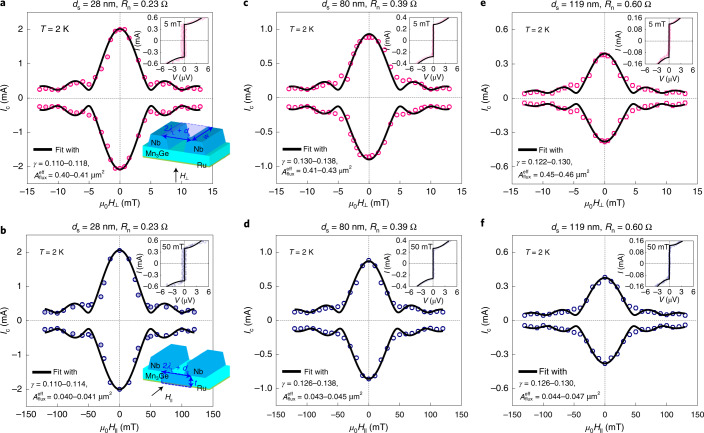
Magnetic field interference patterns reflecting spatial quantum interference. **a**,**c**,**e**, Josephson critical current *I*_c_ versus magnetic field $\mu _0H_ \bot$ plots for the *d*_s_ = 28 (**a**), 80 (**c**) and 119 nm (**e**) Nb/Mn_3_Ge/Nb junctions, taken at a fixed *T* = 2 K. In these measurements, $\mu _0H_ \bot$ is applied perpendicular to the interface plane of the Nb electrodes. The top inset displays the current–voltage *I–V* curve of each junction around zero-order minimum of *I*_c_($\mu _0H_ \bot$). The bottom inset in **a** schematically illustrates the effective junction area $A_{\mathrm{{flux}}}^{\mathrm{{eff}}}$ of magnetic flux penetration, given by $\left( {2\lambda _L + d_{\mathrm{s}}} \right)w$. Here *λ*_*L*_ is the London penetration depth of the Nb electrodes and *w* is the width of the Mn_3_Ge spacer. **b**,**d**,**f**, Data equivalent to **a**,**d**,**e** but for the magnetic field $\mu _0H_{||}$ applied parallel to the interface plane of the Nb electrodes for *d*_s_ = 28 (**b**), 80 (**d**) and 119 nm (**f**). Note that, accordingly, $A_{\mathrm{{flux}}}^{\mathrm{{eff}}}$ changes to $\left( {2\lambda _L + d_{\mathrm{s}}} \right)t$ where *t* is the effective thickness of the Mn_3_Ge spacer.

We next measure the magnetic field interference pattern *I*_c_ (*μ*_0_*H*) in Fig. 3a–f, from which one can evaluate the transverse uniformity^21^ of *I*_c_ across the Mn_3_Ge barrier. For all *d*_s_ = 28, 80 and 119 nm devices, *I*_c_ is strongly modulated by applying a small (modest) external field $\mu _0H_ \bot$ < 15 mT ($\mu _0H_{||}$ < 150 mT) perpendicular (parallel) to the interface plane of Nb electrodes. This excludes a short circuit between the neighbouring Nb electrodes and confirms a genuine Josephson effect^21^. Note that if a short exists, *I*_c_ would be almost independent of $\mu _0H_ \bot$ ($\mu _0H_{||}$) for such a small (modest) field range, as presented in the Supplementary Text. The zero-order maximum of *I*_c_ is obtained around zero applied field $\mu _0H_ \bot$ = $\mu _0H_{||}$ = 0 without a detectable hysteresis, which indicates a vanishingly small spontaneous magnetization and is consistent with features of the AFM spacer^25,26^.

For a single rectangular JJ, taking into account a non-uniform supercurrent density distribution from structural fluctuations^21^ of the barrier, the sinusoidal position-dependent superconducting phase by the enclosed magnetic flux Φ under application of *μ*_0_*H* gives rise to a characteristic modulation of *I*_c_ (ref. ^21^). often referred to as a single-slit Fraunhofer diffraction pattern, $I_{\mathrm{c}}\left( {\mu _0H} \right) = I_{\mathrm{c}}\sqrt {{\mathrm{sinc}}\left( {\frac{{\Phi}}{{{\Phi}_0}}} \right)^2 + \gamma ^2\left[ {1 - {\mathrm{sinc}}\left( {\frac{{\Phi}}{{{\Phi}_0}}} \right)^2} \right]}$. Here ${\Phi} = \mu _0HA_{\mathrm{{flux}}}^{\mathrm{{eff}}}$ and $A_{\mathrm{{flux}}}^{\mathrm{{eff}}}$ is the effective junction area of magnetic flux penetration that is given by $\left( {2\lambda _L + d_{\mathrm{s}}} \right)w$ for $\mu _0H_ \bot$ (bottom inset of Fig. 3a) or $\left( {2\lambda _L + d_{\mathrm{s}}} \right)t$ for $\mu _0H_{||}$ (bottom inset of Fig. 3b) and *λ*_*L*_ is the London penetration depth (130 nm at 2 K)^31^ of 50 nm thick Nb electrodes. *w*(*t*) is the width (effective thickness) of the Mn_3_Ge spacer and ${\Phi}_0 = \frac{h}{{2e}}$ is the magnetic flux quantum. *γ* is a measure of the supercurrent non-uniformity^21^. Best fits to the $I_{\mathrm{c}}\left( {\mu _0H_ \bot } \right)$ and $I_{\mathrm{c}}\left( {\mu _0H_{||}} \right)$ data using this formula give $\gamma = 0.110 - 0.138$, $\left( {2\lambda _L + d_{\mathrm{s}}} \right)w$ = 0.40–0.46 µm^2^ and $\left( {2\lambda _L + d_{\mathrm{s}}} \right)t$ = 0.040–0.047 µm^2^, respectively. We then find *w* = 1.2–1.4 µm and *t* = 120–140 nm, which are close to the actual dimensions of our devices. Rather, monotonic *I*_c_(*μ*_0_*H*) interference patterns with less clear minima (Fig. 3a–f) for our JJs are likely because the position-dependent phase modulation deviates from the sinusoidal form due to the complicated magnetization reversal process^32^ of cluster octupole domains of the chiral AFM^33^, each of which induces a tiny uncompensated magnetization, and thereby the locally varying *µ*_0_*H*-dependent internal phase^32^.

**Fig. 4:**
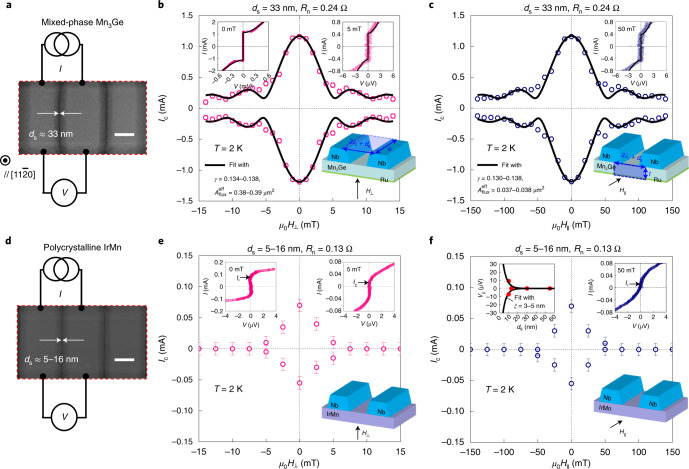
Dependence of Josephson supercurrents on the degree of chiral non-collinearity. **a**, Scanning electron micrograph of the fabricated Nb/Mn_3_Ge/Nb lateral JJ, where the Mn_3_Ge spacer is a mixed phase of tetragonal *D*0_22_ and hexagonal *D*0_19_ (Supplementary Text). The scale bar (**a**,**d**) indicates 0.5 µm. **b**, Magnetic field interface pattern *I*_c_($\mu _0H_ \bot$) when the magnetic field $\mu _0H_ \bot$ is applied perpendicular to the interface plane of Nb electrodes (bottom inset). The top left and right insets display the current–voltage *I–V* curves of the JJ, taken around zero-order maximum and zero-order minimum of *I*_c_($\mu _0H_ \bot$), respectively. **c**, Data equivalent to **b** but for the magnetic field $\mu _0H_{||}$ applied parallel to the interface plane of the Nb electrodes (bottom inset). **d**,**e**,**f**, Data equivalent to **a**,**b**,**c** but for the Nb/IrMn/Nb junction with a much shorter *d*_s_ = 5–16 nm, in which the IrMn spacer is polycrystalline (Supplementary Text). Scanning electron micrograph (**d**), magnetic field interface patterns (**e**,**f**) and data equivalent to **b**,**c**. The left inset in **f** exhibits the characteristic voltage *V*_c_ = *I*_c_*R*_n_ as a function of *d*_s_, from which the decay length scale of the Josephson coupling through the IrMn spacer is determined to the first order (see Supplementary Text for details).

To prove that the chiral non-collinear antiferromagnetic structure, directly linked to the Berry curvature in *k*-space, is responsible for the observed long-range supercurrents, we replace the single-phase hexagonal *D*0_19_-Mn_3_Ge spacer with either a mixed phase of tetragonal *D*0_22_- and hexagonal *D*0_19_-Mn_3_Ge (Fig. 4a–c), or a polycrystalline collinear AFM IrMn (Fig. 4d–f). As presented in the Supplementary Text, the mixed-phase Mn_3_Ge reveals a large zero-field anomalous Hall effect (AHE) comparable to the bulk single crystal^3,4^, ensuring that it still hosts, to a large extent, triangular chiral antiferromagnetic domains relevant to a non-trivial topology^1–5^. In contrast, no AHE is detected in the polycrystalline IrMn, as expected for topologically trivial antiferromagnetic ground states^16^.

The most noteworthy result is that in the presence of chiral non-collinearity (equivalently, non-zero Berry phase), long-ranged Josephson supercurrents are established even in the mixed-phase Mn_3_Ge (Fig. 4b,c) whereas this long-range effect almost disappears when the spin arrangements of the AFM spacer are topologically trivial (Fig. 4e,f). This points unambiguously to a topological origin of Josephson coupling in the chiral AFM Mn_3_Ge, which should be robust against structural disorder and impurity scattering. Note that the *ξ* value for the IrMn spacer is estimated to first order to be 3–5 nm (left inset of Fig. 4f and Supplementary Text) and this short-ranged Josephson coupling through the collinear AFM IrMn is in good agreement with previous reports on vertical JJs with γ−Fe_50_Mn_50_ (ref. ^25^) or Cr (ref. ^26^) spacers. An intuitive explanation of these results is as follows. The 120° non-collinear arrangements of Mn magnetic moments on the atomic-scale in real-space convert spin-unpolarized singlet Cooper pairs (*S* = 0) to spin-zero triplets (*S* = 1, *m*_s_ = 0). The converted spin-zero triplets (*S* = 1, *m*_s_ = 0) in motion then experience fictitious magnetic fields (as large as roughly 100 tesla)^2–4^ associated with the Berry curvature^14^ in *k*-space and rotate to form spin-polarized triplets (*S* = 1, *m*_s_ = ±1), which are able to penetrate much deeper^6–8^. Here, the fictitious magnetic fields rooted in the chiral non-collinear spin texture^2–4^ play a crucial role in changing the quantization axis of the spin-zero triplets (*S* = 1, *m*_s_ = 0) to be converted into the spin-polarized triplets (*S* = 1, *m*_s_ = ±1). None of the above are present in the IrMn spacer, accounting for its short-range nature of Josephson coupling^6–8,24,25^.

The essential ingredient for the realization of the long-range spin-triplet proximity effect^6–8^ is the presence of a magnetically inhomogeneous ferromagnet/superconductor interface (often called a spin-active interface), which results in subsequent spin-mixing and spin-rotation processes^9–14^, and so far, the spin-triplet proximity effect has been experimentally observed in various JJs with half-metallic ferromagnet (CrO_2_)^13^, intrinsically inhomogeneous conical ferromagnet (Ho)^11^ and non-collinear magnetic heterostructures (PdNi or CuNi)^12^. The CrO_2_-based lateral JJs have revealed an exceptionally long decay length of 0.3‒1 µm (ref. ^13^), which is supported by its half-metallicity, albeit uncertainty in the nature of the magnetic inhomogeneity. The notable aspect of the present study is that instead of the nanometre-scale inhomogeneity of ferromagnetic materials, an atomic-scale non-collinear AFM (in combination with fictitious magnetic fields) is exploited to generate the spin-triplet correlation that can extend over 155‒160 nm, comparable to the CrO_2_-based JJs^13^.

We have experimentally demonstrated that lateral Josephson supercurrents through a triangular chiral AFM Mn_3_Ge (refs. ^3–5^) are long-ranged, which is a key aspect of proximity-induced spin-polarized triplet pairing states^6–14^. Although detailed theories, covering the triplet superconductivity and Berry curvature, need to be developed for a quantitative description, our results provide the experimental indication of topologically generated triplet pairing states via a chiral non-collinear AFM, which can potentially resolve the outstanding issues raised in conventional ferromagnet-based triplet JJs^6–14^. Last but not least, the characteristic decay length of Josephson supercurrents in our chiral AFM is found, not to be limited by hitherto believed spin-diffusion lengths, but rather hinting at topologically protected triplet supercurrents.

## Methods

### Sample preparation and characterization

All the thin films were grown by d.c. magnetron sputtering in a homemade ultra-high vacuum system with a base pressure of 1 × 10^−9^ Torr. The single-phase hexagonal *D*0_19_-Mn_3_Ge(0001) thin film was grown epitaxially on a Ru-buffered Al_2_O_3_(0001) substrate. A 5 nm thick Ru buffer layer was first sputtered at 450 °C with a sputter power of 15 W and at an Ar pressure of 3 mTorr. The Mn and Ge were subsequently codeposited from elemental sputter targets on the Ru(0001) buffer layer at 500 °C and at an Ar pressure of 3 mTorr. The optimized sputter powers were 31 and 10 W for Mn and Ge, respectively. The final composition was determined ex situ by Rutherford backscattering spectrometry to be Mn_3.2_Ge, where the excess Mn helps to stabilize the hexagonal *D*0_19_ phase^28^. To study the ground-state chiral non-collinearity dependence (Fig. 4), the mixed phase of tetragonal *D*0_22_- and hexagonal *D*0_19_-Mn_3_Ge($11\bar 20$) film was prepared using the same growth procedure but on a Ru-buffered sapphire Al_2_O_3_$(1\bar 102)$ substrate and the polycrystalline thin film of collinear AFM IrMn was sputtered at 27 °C on a SiO_2_(25 nm)/Si substrate. The used sputter powers were 15 and 30 W for Ir and Mn, respectively. We note here that the thickness of Mn_3_Ge and IrMn films was fixed at 40 nm that is much larger than their singlet coherence length (a few nanometres)^24–26^. So the singlet-pair-mediated Josephson coupling through the ultrathin Ru buffer layer of Nb/Mn_3_Ge/Nb junctions is unlikely (see the main text for a detailed discussion). Using these unpatterned films, we performed magneto-transport measurements in a Van der Pauw geometry to investigate the Berry curvature-driven AHE^1–5^ (Supplementary Text). To probe AFM ordering, indirectly from exchange-biased magnetic hysteresis curve measurements (Supplementary Text), a 10 nm thick Ni_8_Fe_2_ layer was sputtered on top of the Mn_3_Ge and IrMn film. All these films were capped with a 1 nm thick AlO_x_ layer to prevent oxidation.

### Device fabrication

To fabricate the lateral JJs (Fig. 1a), we first defined a central AFM track with a lateral dimension of 1.5 × 50 μm^2^ using optical lithography and Ar-ion beam etching. We then defined Au (80 nm)/Ru (2 nm) electrical leads and bonding pads, which were deposited by Ar-ion beam sputtering. Subsequently, we defined multiple Nb electrodes with an active lateral dimension of 1.0 × 1.5 μm^2^ on top the AFM track via electron-beam lithography and lift-off steps. The 50-nm thick Nb electrodes were grown by Ar-ion beam sputtering at a pressure of 1.5 × 10^–4^ mbar and they were edge-to-edge separated by 28–119 nm from each other. Before sputtering the Nb electrodes, the Al_2_O_3_ capping layer and Au surface were Ar-ion beam etched away to make possible direct metallic electrical contacts.

### Transport measurement

We measured current–voltage *I–V* curves of the fabricated JJs (Fig. 1a) with a four-probe configuration in a Quantum Design Physical Property Measurement System using a Keithley 6221 current source and a Keithley 2182A nanovoltmeter. The zero-field *I–V* curves (Fig. 2a–f) were measured across the superconducting transition of Nb electrodes. The Josephson critical current *I*_c_ and the normal-state zero-bias resistance *R*_n_ of each JJ (Fig. 2g) were determined by fitting the measured *I–V* curves with the standard formula for overdamped junctions^21^, $V(I) = \frac{I}{{|I|}}R_{\mathrm{n}}\sqrt {I^2 - I_{\mathrm{c}}^2}$. As the *I–V* curves were slightly shifted horizontally, we defined *I*_c_ values in positive and negative current directions. We obtained the magnetic field interference patterns *I*_*c*_ (*μ*_0_*H*) (Figs. 3a–f and 4b,c) by repeating the *I–V* measurements at *T* = 2 K at the applied magnetic field $\mu _0H_ \bot$ ($\mu _0H_{||}$) perpendicular (parallel) to the interface plane of Nb electrodes. We note here that no significant change in the magnetic interference patterns between zero-field-cooled and field-cooled (1 T) conditions for our JJs with the single-phase Mn_3_Ge(0001) spacer has been observed. This supports that the chiral non-collinear atomic-scale AFM spin arrangements (as strong as a few hundred tesla at least) are indeed responsible for the observed long-range Josephson coupling in our system. For the same reason, asymmetric hysteretic behaviour in *R*-$\mu _0H_ \bot$ curves of our chiral AFM JJs, reflecting a link between the magnetic ordering and the superconducting state, is weak, but evident, further supporting the spin-polarized triplet pairing interpretation (Supplementary Text).

## Online content

Any methods, additional references, Nature Research reporting summaries, source data, extended data, supplementary information, acknowledgements, peer review information; details of author contributions and competing interests; and statements of data and code availability are available at 10.1038/s41563-021-01061-9.

## Supplementary information

Supplementary Information Supplementary Text, Figs. 1–8 and references.

## Data Availability

The data used in this paper are available from the corresponding authors upon reasonable request.

## References

[1] Machida Y, Nakatsuji S, Onoda S, Tayama T, Sakakibara T (2010). Time-reversal symmetry breaking and spontaneous Hall effect without magnetic dipole order. Nature.

[2] Nakatsuji S (2015). Large anomalous Hall effect in a non-collinear antiferromagnet at room temperature. Nature.

[3] Nayak AK (2016). Large anomalous Hall effect driven by a nonvanishing Berry curvature in the noncolinear antiferromagnet Mn_3_Ge. Sci. Adv..

[4] Kiyohara N, Tomita T, Nakatsuji S (2016). Giant anomalous Hall effect in the chiral antiferromagnet Mn_3_Ge. Phys. Rev. Applied.

[5] Soh J-R (2020). Ground-state magnetic structure of Mn_3_Ge. Phys. Rev. B.

[6] Linder J, Robinson JWA (2015). Superconducting spintronics. Nat. Phys..

[7] Eschrig M (2015). Spin-polarized supercurrents for spintronics: a review of current progress. Rep. Prog. Phys..

[8] Birge NO (2018). Spin-triplet supercurrents in Josephson junctions containing strong ferromagnetic materials. Phil. Trans. R. Soc. A.

[9] Bergeret FS, Volkov AF, Efetov KB (2001). Long-range proximity effects in superconductor-ferromagnet structures. Phys. Rev. Lett..

[10] Houzet M, Buzdin AI (2007). Long range triplet Josephson effect through a ferromagnetic trilayer. Phys. Rev. B.

[11] Robinson JWA, Witt JDS, Blamire MG (2010). Controlled injection of spin-triplet supercurrents into a strong ferromagnet. Science.

[12] Khaire TS, Khasawneh MA, Pratt WP, Birge NO (2010). Observation of spin-triplet superconductivity in Co-based Josephson junctions. Phys. Rev. Lett..

[13] Keizer RS (2006). A spin triplet supercurrent through the half-metallic ferromagnet CrO_2_. Nature.

[14] Cottet A (2011). Inducing odd-frequency triplet superconducting correlations in a normal metal. Phys. Rev. Lett..

[15] Xiao D, Chang M-C, Niu Q (2010). Berry phase effects on electronic properties. Rev. Mod. Phys..

[16] Zhou J (2019). Large spin-orbit torque efficiency enhanced by magnetic structure of collinear antiferromagnet IrMn. Sci. Adv..

[17] Jeon K-R (2018). Enhanced spin pumping into superconductors provides evidence for superconducting pure spin currents. Nat. Mater..

[18] Banerjee N (2018). Controlling the superconducting transition by spin-orbit coupling. Phys. Rev. B.

[19] Bergeret FS, Tokatly IV (2014). Spin-orbit coupling as a source of long-range triplet proximity effect in superconductor-ferromagnet hybrid structures. Phys. Rev. B.

[20] Arras E, Caliste D, Deutsch T, Lançon F, Pochet P (2011). Phase diagram, structure, and magnetic properties of the Ge-Mn system: a first-principles study. Phys. Rev. B.

[21] Barone, A. & Paterno, G. *Physics and Applications of the Josephson Effect* 2nd edn (John Wiley & Sons, 1982).

[22] Likharev KK (1979). Superconducting weak links. Rev. Mod. Phys..

[23] Rusanov AY, Hesselberth MBS, Aarts J (2004). Depairing currents in superconducting films of Nb and amorphous MoGe. Phys. Rev. B.

[24] Krivoruchko VN (1996). Upper critical fields of the superconducting state of a superconductor-antiferromagnetic metal superlattice. J. Exp. Theor. Phys..

[25] Bell C (2003). Proximity and Josephson effects in superconductor/antiferromagnetic Nb/γ−Fe_50_Mn_50_ heterostructures. Phys. Rev. B.

[26] Weides M, Disch M, Kohlstedt H, Bürgler DE (2009). Observation of Josephson coupling through an interlayer of antiferromagnetically ordered chromium. Phys. Rev. B.

[27] Wang X (2021). Robust anomalous Hall effect and temperature driven Lifshitz transition in Weyl semimetal Mn_3_Ge. Nanoscale.

[28] Hong D (2020). Large anomalous Nernst and inverse spin-Hall effects in epitaxial thin films of kagome semimetal Mn_3_Ge. Phys. Rev. Mater..

[29] Muduli PK (2019). Evaluation of spin diffusion length and spin Hall angle of the antiferromagnetic Weyl semimetal Mn_3_Sn. Phys. Rev. B.

[30] Flokstra MG (2016). Remotely induced magnetism in a normal metal using a superconducting spin-valve. Nat. Phys..

[31] Gubin AI, Il’in KS, Vitusevich SA (2005). Dependence of magnetic penetration depth on the thickness of superconducting Nb thin films. Phys. Rev. B.

[32] Börcsök B, Komori S, Buzdin AI, Robinson JWA (2019). Fraunhofer patterns in magnetic Josephson junctions with non-uniform magnetic susceptibility. Sci. Rep..

[33] Liu J, Balents L (2017). Anomalous Hall effect and topological defects in antiferromagnetic Weyl semimetals: Mn_3_Sn/Ge. Phys. Rev. Lett..

