# Supplementary material for: Long-range supercurrents through a chiral non-collinear antiferromagnet in lateral Josephson junctions
Source: Nat Mater. 2021 Aug 5;20(10):1358–63. doi: 10.1038/s41563-021-01061-9 (PMC8463295; doi:10.1038/s41563-021-01061-9)
Supplement: Supplementary file 1 — Supplementary Text, Figs. 1–8 and references. [file 41563_2021_1061_MOESM1_ESM.pdf]

nature portfolio

https://doi.org/10.1038/s41563-021-01061-9

Supplementary information

Long-range supercurrents through a chiral non-collinear antiferromagnet in lateral Josephson junctions

In the format provided by the authors and unedited

Supplementary Information

Long-range supercurrents through a chiral non-collinear antiferromagnet in lateral Josephson junctions

Kun-Rok Jeon[1*†], Binoy Krishna Hazra[1†], Kyungjune Cho[1], Anirban Chakraborty[1], Jae-Chun Jeon[1], Hyeon Han[1], Holger L. Meyerheim[1], Takis Kontos[2] and Stuart S. P. Parkin[1*]

[1]*Max Planck Institute of Microstructure Physics, Weinberg 2, 06120 Halle (Saale), Germany*

[2]*Laboratoire de Physique de l'Ecole normale supérieure, ENS, Université PSL, CNRS, Sorbonne Université, Université de Paris, F-75005 Paris, France*

[†]These authors contributed equally to this work.

*To whom correspondence should be addressed: jeonkunrok@gmail.com, stuart.parkin@halle-mpi.mpg.de

This PDF file includes:

Supplementary Text

Figs. *S1* to *S8*

References (*S1-S33*)

Section S1. Structural analysis of the chiral non-collinear AFM Mn_3Ge and the collinear AFM IrMn.

In this section, we describe the detailed structural investigation of Mn_3Ge, and IrMn films using X-ray diffraction (XRD). Figure S1a shows the specular $\theta-2\theta$ longitudinal XRD scan for a 40 nm Mn_3Ge (0001) film grown on Ru buffered (5 nm) Al_2O_3 (0001) oriented substrate. Since the Ru (0002) $\{2\theta = 42.15\}$[S1] and the Mn_3Ge (0002) $\{2\theta = 41.82\}$[S2] reflections are nearby, it is very difficult to separate them from each other in the $\theta-2\theta$ scan. However, it is evident from the inset of Fig. S1a that the (0002) reflection of Mn_3Ge/Ru bilayer becomes sharp and its intensity is also enhanced as compared to the bare Ru film. In the entire $\theta-2\theta$ scan, only the Ru (0002), Ru (0004), Mn_3Ge (0002) and Mn_3Ge (0004) reflections are observed, which indicates the single-crystalline nature and the (0001) oriented Mn_3Ge film. Furthermore, we have investigated non-specular reflections by transverse phi ϕ scans for the Al_2O_3 $\{10\bar{1}4\}$, Ru $\{20\bar{2}3\}$ and the Mn_3Ge $\{20\bar{2}3\}$ reflections (Fig. S1b). The Al_2O_3 $\{10\bar{1}4\}$ diffraction pattern exhibits a threefold symmetry, whereas a sixfold symmetry is observed from the Ru $\{20\bar{2}3\}$ and Mn_3Ge $\{20\bar{2}3\}$ reflection. The lattice vectors of the Ru layer are rotated by 30° with respect to those of the Al_2O_3 substrate while they are aligned with those of Mn_3Ge. Unlike the single-crystalline Mn_3Ge (0001) film, the 40 nm Mn_3Ge $(11\bar{2}0)$ film which is sputtered on 5 nm Ru buffered Al_2O_3 $(1\bar{1}02)$ substrate is found to be a mixed phase composed of an epitaxial $D0_{19}$ Mn_3Ge $(11\bar{2}0)$ (hexagonal phase) and a polycrystalline $D0_{22}$ Mn_3Ge (tetragonal phase) (Fig. S1c). Hence we call this the mixed-phase Mn_3Ge $(11\bar{2}0)$ film. In addition, the Ru (0002) reflection overlaps with the tetragonal Mn_3Ge (101) whereas the Ru $(11\bar{2}0)$ reflection overlaps with both hexagonal Mn_3Ge $(22\bar{4}0)$ and tetragonal Mn_3Ge (112) reflections. These overlapped peaks in the mixed-phase Mn_3Ge $(11\bar{2}0)$ film are distinguished based on the comparison of peak intensity and full-width-at-half-maxima (FWHM) with the bare Ru film sputtered on Al_2O_3 $(1\bar{1}02)$ substrate. The IrMn film which was deposited at room temperature on SiO_2 (25

nm)/Si (001) substrate is found to be polycrystalline (Fig. S1d) in nature. In order to estimate the amount of the tetragonal $D0_{22}$ phase relative to the hexagonal $D0_{19}$ phase in the mixed phase Mn_3Ge film, we carry out additional X-ray diffraction experiments using a Gallium-Jet X-ray source operated at 70 keV and 100 W emitting Ga-Kα radiation (λ = 1.3414 Å). Reflections are recorded by using a two-dimensional pixel detector and a six-circle diffractometer operated in the z-axis mode, where the primary beam is incident under grazing angle (μ = 1°) to the sample surface. Figures S1e and S1f show transverse ϕ scans (around the surface normal) across the tetragonal $D0_{22}$ Mn_3Ge (101) reflection and the hexagonal $D0_{19}$ Mn_3Ge (201) reflection, respectively. First, the observation of three groups of reflections separated by 90 degrees indicates the presence of several crystalline domains of the tetragonal structure whose lattice parameters were deduced to be a = 2.689 Å and c = 3.618 Å corresponding to the bulk values[S3].

On the other hand, for the hexagonal $D0_{19}$ phase, only one set of symmetry-equivalent reflections is observed, *e.g.* the zoom-in (201) reflection is shown in Fig. S1f. The relative volume ratio between the $D0_{22}$ and $D0_{19}$ phases of the mixed-phase Mn_3Ge $(11\bar{2}0)$ film can be estimated by considering the integrated intensities $I(hkl)$ of the reflections,

$$I(hkl) \propto \frac{|F(hkl)|^2 V_x}{(v)^2}$$

where $|F(hkl)|^2$ and V_x (v) represent the amplitude of structure factors and the volume of crystal (unit cell) for each phase, respectively. Using the calculated $|F(hkl)|^2$ values for the respective structures and reflections (Figs. S1e and S1f), we obtain $V_x(D0_{22})$: $V_x(D0_{19})$ ≈ 2 : 3, *i.e.* the volume fraction of the hexagonal phase is 1.5 times larger than that of the tetragonal phase. In contrast, for the single-phase Mn_3Ge (0001) film, $V_x(D0_{22})$: $V_x(D0_{19})$: ≈ 1 : 50 is estimated (not shown), confirming a vanishingly small volume fraction of the tetragonal phase.

The atomic force micrograph (Fig.1Sg) reveals that Mn_3Ge (0001) film is quite smooth (root-mean-square roughness R_{rms} = 1 nm) and the mixed phase Mn_3Ge film is relatively rough

(R_{rms} = 3 nm). The rms roughness for the IrMn film is found be 1 nm. We note that AlO_x capping layer hinders accurate measurement of the rms roughness of each film.

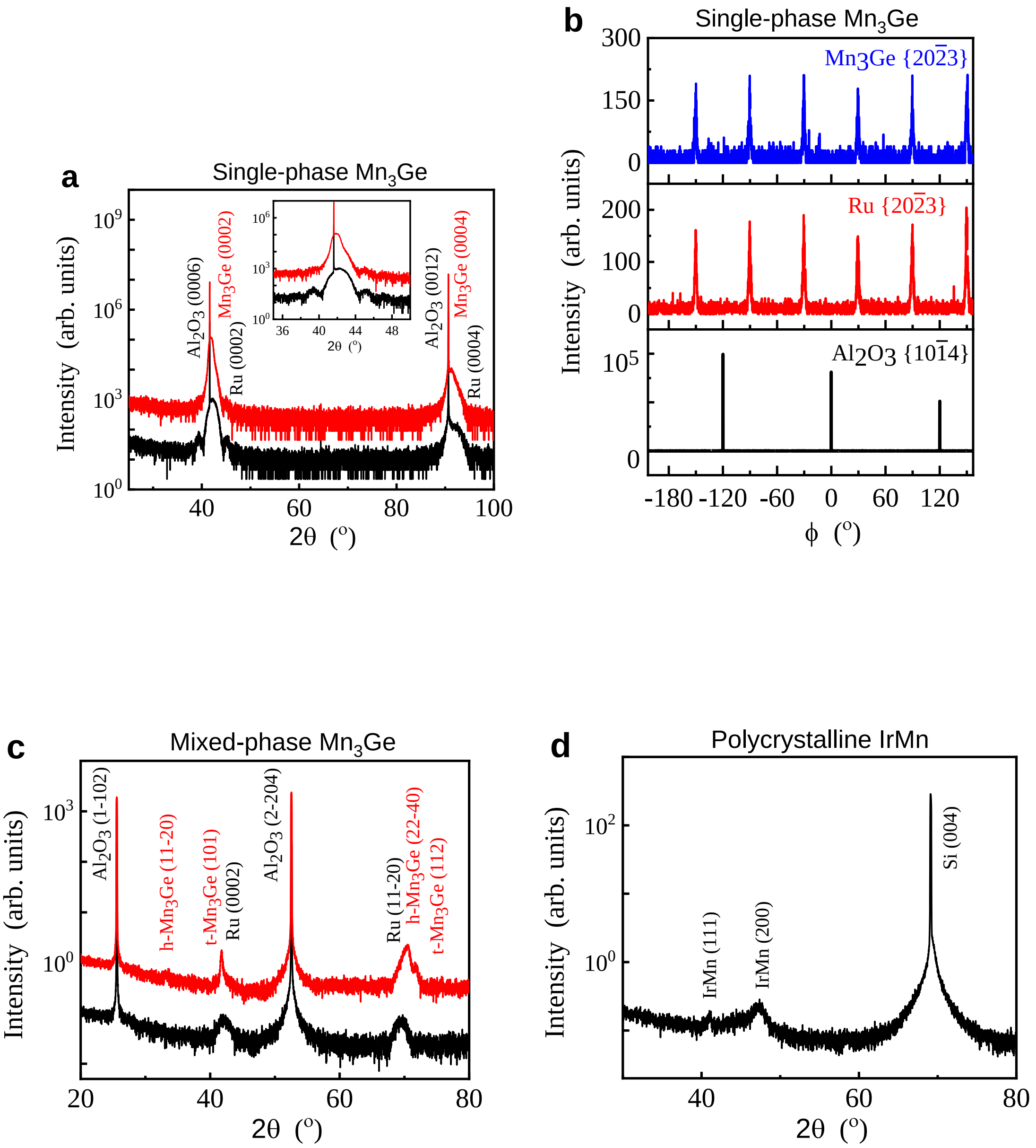

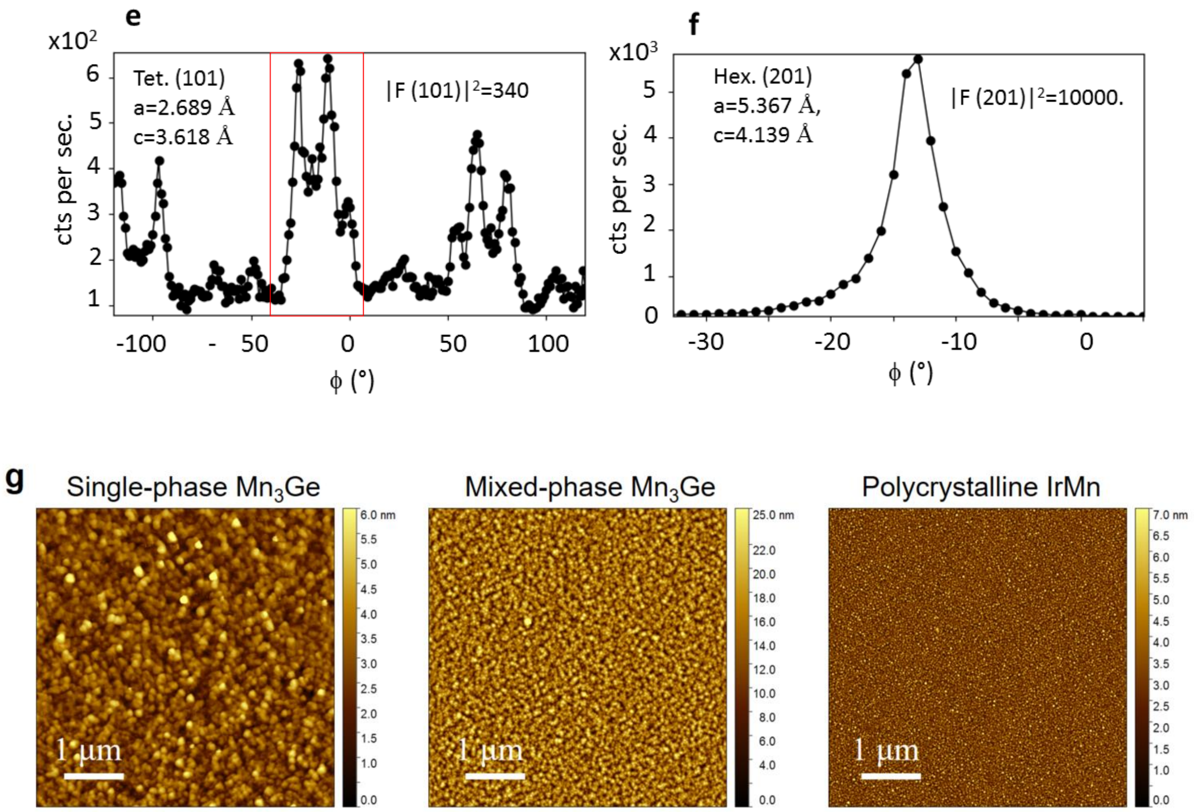

Figure S1. a, Specular θ-2θ XRD pattern of single-crystalline Ru (0001) (black symbol) and single-phase Mn_3Ge(0001)/Ru (red symbol) films grown on Al_2O_3 (0001) substrates. Inset shows the magnified view θ-2θ scan in the range of 35°-50°. **b,** Phi ϕ scan across the Al_2O_3 $\{10\bar{1}4\}$, Ru $\{20\bar{2}3\}$, and Mn_3Ge $\{20\bar{2}3\}$ reflections for the same film. **c,** X-ray diffraction pattern of the mixed-phase Mn_3Ge/Ru film (red color) grown on Al_2O_3 $(1\bar{1}02)$. **d,** Diffraction pattern of the polycrystalline IrMn film grown on SiO_2(25 nm)/Si substrate. **e,** Wide angle ϕ-scan across the (101) reflection of the tetragonal $D0_{22}$ Mn_3Ge phase, showing several groups of 90° symmetry-equivalent reflections, each of which originates from a different crystalline domain. This is in accordance with the fourfold rotational symmetry of the tetragonal $D0_{22}$ Mn_3Ge phase. Lattice parameters for *a* and *c* axes are given in the inset. **f,** Narrow angle ϕ-scan across one of the six symmetry-equivalent (201) reflections of the hexagonal $D0_{19}$ Mn_3Ge phase. Integrated intensities in **e** and **f** were used to estimate the relative abundance of the mixed-phase Mn_3Ge $(11\bar{2}0)$ sample using the calculated structure factor magnitudes for the respective reflections (see supplementary text for details). **g,** Atomic force micrographs of the single-phase Mn_3Ge (0001), mixed-phase Mn_3Ge, and polycrystalline IrMn films over 5×5 μm^2 scan area (from left to right respectively).

Section S2. Magnetic properties of the chiral non-collinear AFM Mn_3Ge and the collinear AFM IrMn.

We discuss the magnetic properties for all samples. The kagome planes of $D0_{19}$ Mn_3Ge bulk crystal are in the (0001) plane. In case of the single-phase $D0_{19}$ Mn_3Ge (0001) film, the kagome planes are thus oriented perpendicular to the [0001] growth direction and a small uncompensated magnetic moment lies in the film plane. On the other hand, for the mixed-phase $D0_{19}$ Mn_3Ge $(11\bar{2}0)$, the kagome planes are parallel to the $[11\bar{2}0]$ preferential growth direction (*i.e.* perpendicular to the film plane) and so the small remanent magnetic moment exists in the out-of-plane direction.

Magnetization versus in-plane magnetic field $M(\mu_0 H_{\parallel})$ curves [the black (blue) represents raw (corrected) data after subtracting diamagnetic background] illustrate that the single-phase $D0_{19}$ Mn_3Ge (0001) film possesses a small in-plane magnetization of 42 emu/cm^3 and 80 emu/cm^3 at 300 K and 2 K, respectively (Figs. S2a and S2b). These M values are close to the reported magnetization of $D0_{19}$ Mn_3Ge film[S5] and $D0_{19}$ Mn_3Sn film[S6] whereas the calculated magnetic moment per unit Mn atom (~ $0.07\mu_B$) is found to be larger than the bulk Mn_3Ge crystal[S7]. This discrepancy can be of structural origin since quantitative XRD structure refinements for the single-phase films of Mn_3Ge indicate the presence of chemical and structural disorders involving an excess of Mn relative to the Mn_3Ge stoichiometry and the occupation of vacancies at different lattice sites of space group $P6_3/mmc$ by Ge atoms.

To determine the Néel temperature $T_{Néel}$, we have measured the magnetization as a function of temperature with a small in-plane-field-cooled condition ($\mu_0 H_{\parallel}$ = 10 mT, Fig. S2c). The found $T_{Néel}$ value of ~ 410 K by extrapolating the magnetization to zero is slightly higher than the reported bulk value[S7,S15]. This is likely duet to a slight different composition[S14] in our film compare to the bulk crystal [S7,S15].

Furthermore, we have carried out exchange bias measurements on Py (10 nm)/single-phase Mn_3Ge (0001) (40 nm) bilayer at 2 K with -1 T magnetic-field-cooled condition from 400 K. A clear shift $\mu_0 H_{EB}$ of the hysteresis loop (Fig. S2d) along the positive magnetic field direction manifests the finite exchange bias of $\mu_0 H_{EB}$ = 3.8 mT. The coercive field $\mu_0 H_c$ of the bilayer is found to be $\mu_0 H_c$ = 20 mT which is 20 times larger than that of a bare Py film, indicating a strong interlayer exchange coupling across the Py/Mn_3Ge interface. Similar behaviors have been reported recently in Py/Mn_3Ge bilayers[S4,S8], from which one can indirectly probe AFM ordering of the Mn_3Ge film. Note that the observed finite exchange bias demonstrates that the AFM domains of triangular Mn_3Ge are pinned at the interface, resulting in a unidirectional anisotropy[S9] to the neighboring Py layer.

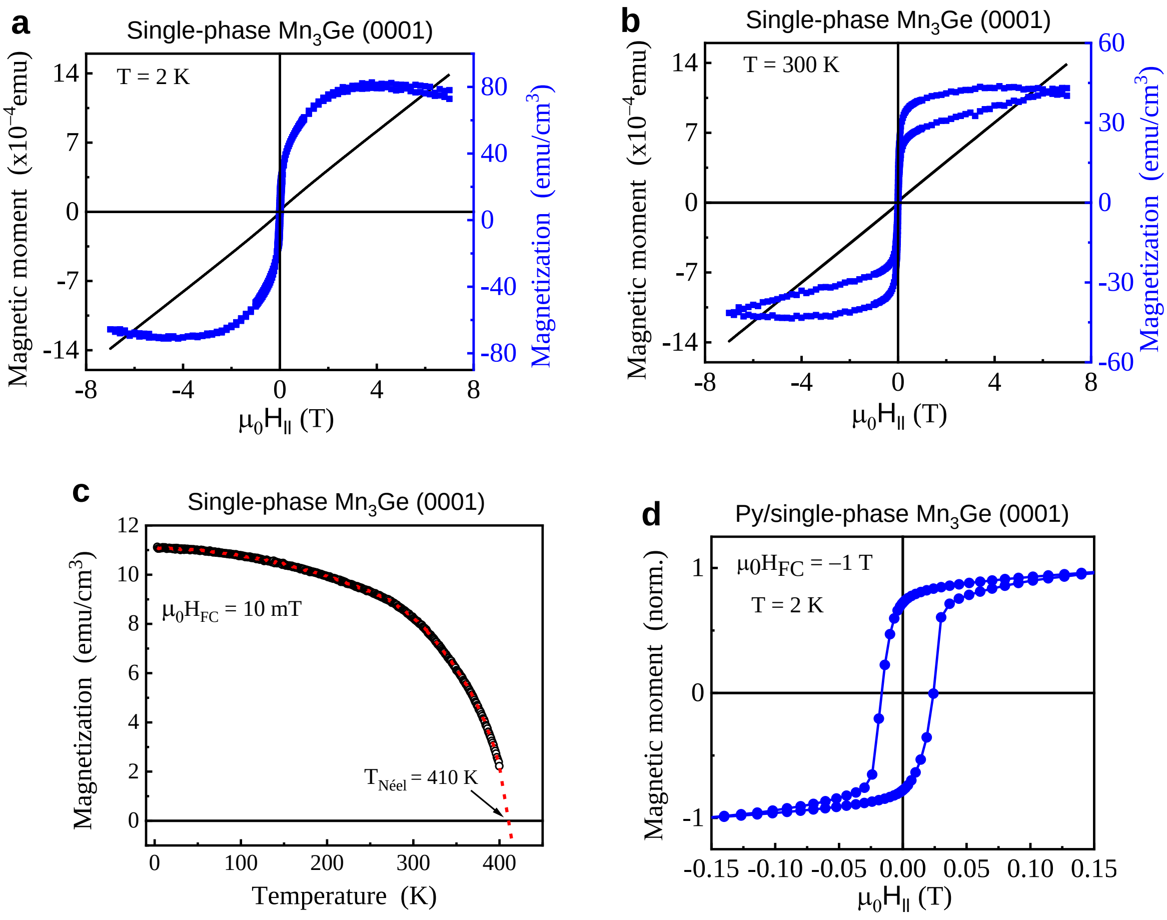

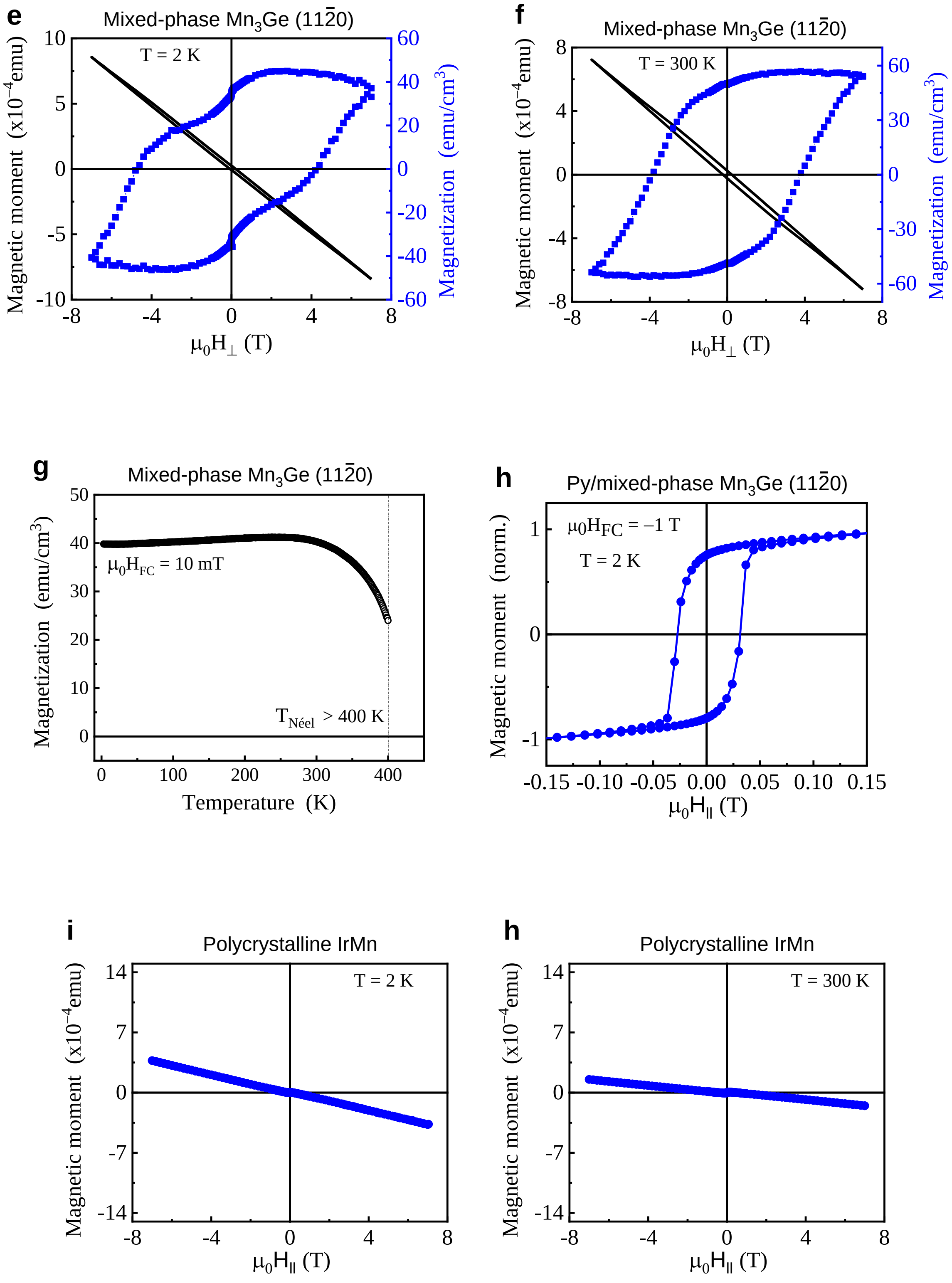
e
Mixed-phase Mn3Ge (112̄0)
T = 2 K
Magnetic moment (x10−4emu)
Magnetization (emu/cm3)
μ0H⊥ (T)
f
Mixed-phase Mn3Ge (112̄0)
T = 300 K
Magnetic moment (x10−4emu)
Magnetization (emu/cm3)
μ0H⊥ (T)
g
Mixed-phase Mn3Ge (112̄0)
μ0HFC = 10 mT
TNéel > 400 K
Magnetization (emu/cm3)
Temperature (K)
h
Py/mixed-phase Mn3Ge (112̄0)
μ0HFC = −1 T
T = 2 K
Magnetic moment (norm.)
μ0H|| (T)
i
Polycrystalline IrMn
T = 2 K
Magnetic moment (x10−4emu)
μ0H|| (T)
h
Polycrystalline IrMn
T = 300 K
Magnetic moment (x10−4emu)
μ0H|| (T)

k

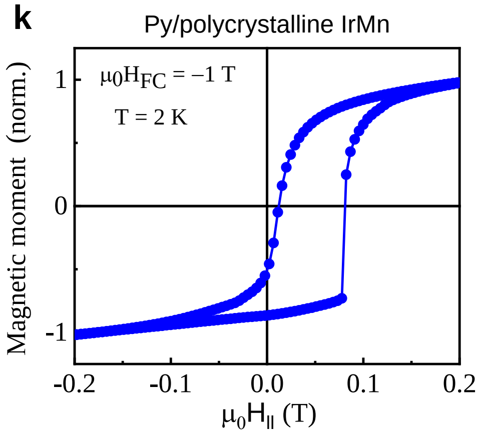

Figure S2. a,b, Magnetic hysteresis *M-H* curves of the single-phase 40 nm Mn_3Ge (0001) film at 300 K and 2 K. Black represents the raw measured data and blue represents the corrected data after subtracting the diamagnetic contribution. **c**, Magnetization as a function of temperature for the single-phase 40 nm Mn_3Ge (0001) film at $\mu_0 H_{\parallel}$ =10 mT with field cooled condition. **d,** *M-H* curve of Py (10 nm)/single-phase Mn_3Ge(0001) (40 nm) bilayer at 2 K with -1 T field cooled condition. Here Py = $Ni_{81}Fe_{19}$. **e,f,** Magnetic hysteresis *M-H* curves of the mixed phase 40 nm Mn_3Ge ($11\bar{2}0$) film at 300 K and 2 K. **g**, Temperature dependence of magnetization for the mixed-phase 40 nm Mn_3Ge ($11\bar{2}0$) film at $\mu_0 H_{\perp}$ =10 mT with field cooled protocol. **h,** *M-H* curve of Py (10 nm)/mixed-phase Mn_3Ge ($11\bar{2}0$) film (40 nm) bilayer at 2 K. **i,j** *M-H* curves of the polycrystalline 40 nm IrMn film at 300 K and 2 K. **k,** *M-H* curve of Py (10 nm)/polycrystalline IrMn (40 nm) bilayer at 2 K.

We next measure $\mu_0 M$ as a function of out-of-plane magnetic field $\mu_0 H_{\perp}$ (‖ [$11\bar{2}0$]) for the mixed-phase Mn_3Ge ($11\bar{2}0$) film. A large coercive field $\mu_0 H_c$ = 3.8 T (4.5 T) and a saturation magnetization M_s = 56 emu/cm^3 (44 emu/cm^3) are obtained at 300 K (2 K) respectively (Figs. 2Se and 2Sf). Such a large coercive field suggests that there exists a finite volume fraction of the tetragonal $D0_{22}$ phase in the mixed-phase Mn_3Ge film. Given that the tetragonal $D0_{22}$ Mn_3Ge is a ferrimagnet having a distinctively large coercive field and small magnetization (~100 emu/cm^3)[S10,S11], M ($\mu_0 H_{\perp}$) data match well XRD results (see Section S1).

No significant change in the saturation magnetization between 2 K and 300 K is observed, implying nontrivial internal coupling between *D0₂₂* Mn_3Ge collinear ferrimagnet and *D0₁₉* Mn_3Ge non-collinear antiferromagnet. Moreover, we have measured the temperature dependent magnetization (Fig. 2Sg) under $\mu_0 H_{\perp}$ = 10 mT field cooled condition which clearly shows a slight shift of $T_{Néel}$ beyond 400 K. This might be because of the presence of a finite $D0_{22}$ Mn_3Ge phase whose Curie temperature T_c is 920 K[S12].

Applying the same measurement protocol as used for the Py (10 nm)/single-phase Mn_3Ge (0001) to a Py (10 nm)/mixed-phase Mn_3Ge $(11\bar{2}0)$ (40nm) bilayer (Fig. S2h), we find $\mu_0 H_{EB}$ = 2 mT and $\mu_0 H_c$ =29.4 mT. These values are comparable to those determined for the Py (10 nm)/single-phase Mn_3Ge bilayer.

The bare polycrystalline IrMn film does not reveal any finite hysteresis detectable at both 300 K and 2 K (Figs. S2i and S2j), as would be expected for a topologically trivial collinear AFM. A strong exchange bias of $\mu_0 H_{EB}$ = 37.5 mT is observed for the IrMn (40 nm)/Py (10 nm) bilayer at 2 K (Fig. S2k) confirms (collinear) AFM ordering of the IrMn film.

Section S3. Electrical properties of the chiral non-collinear AFM Mn_3Ge and the collinear AFM IrMn.

In this section, we outline the temperature-dependent electrical resistivity $\rho_{ch}(T)$ of the Mn_3Ge and IrMn films, which are Hall-bar-patterned (Fig. S3a) on the same sample where several independent Josephson junctions are fabricated. Here $\rho_{ch} = R_{ch}\left(\frac{wt}{l}\right)$, R_{ch} is the channel resistance. w, t and l are the width, thickness and length of the channel, respectively. As in the case of the single- and mixed-phase Mn_3Ge samples, there exists a 5 nm thick Ru buffer layer (see Methods), the estimated values should be considered as effective ones for the resistivity. Note that the effective resistivity of the single-phase Mn_3Ge(40 nm)/Ru(5 nm) bilayer at 8 K (31 μΩ cm, Fig. S4c) is found to be very close to that (26 μΩ cm)

extracted from the $R_n(d_s)$ data (Fig. 3Sf) using the transmission line (TL) theory[S13], $R_n = 2R_i + R_{ch} = \frac{\rho_{ch}}{tw}(2l_t + d_s)$. Here $R_i = \frac{r_i}{wl_t}$, r_i is the resistance-area product of Nb/Mn_3Ge interfaces (1 mΩ μm^2), $l_t = \sqrt{\frac{r_i}{\frac{\rho_{ch}}{t}}}$ is the charge transfer length (13 nm).

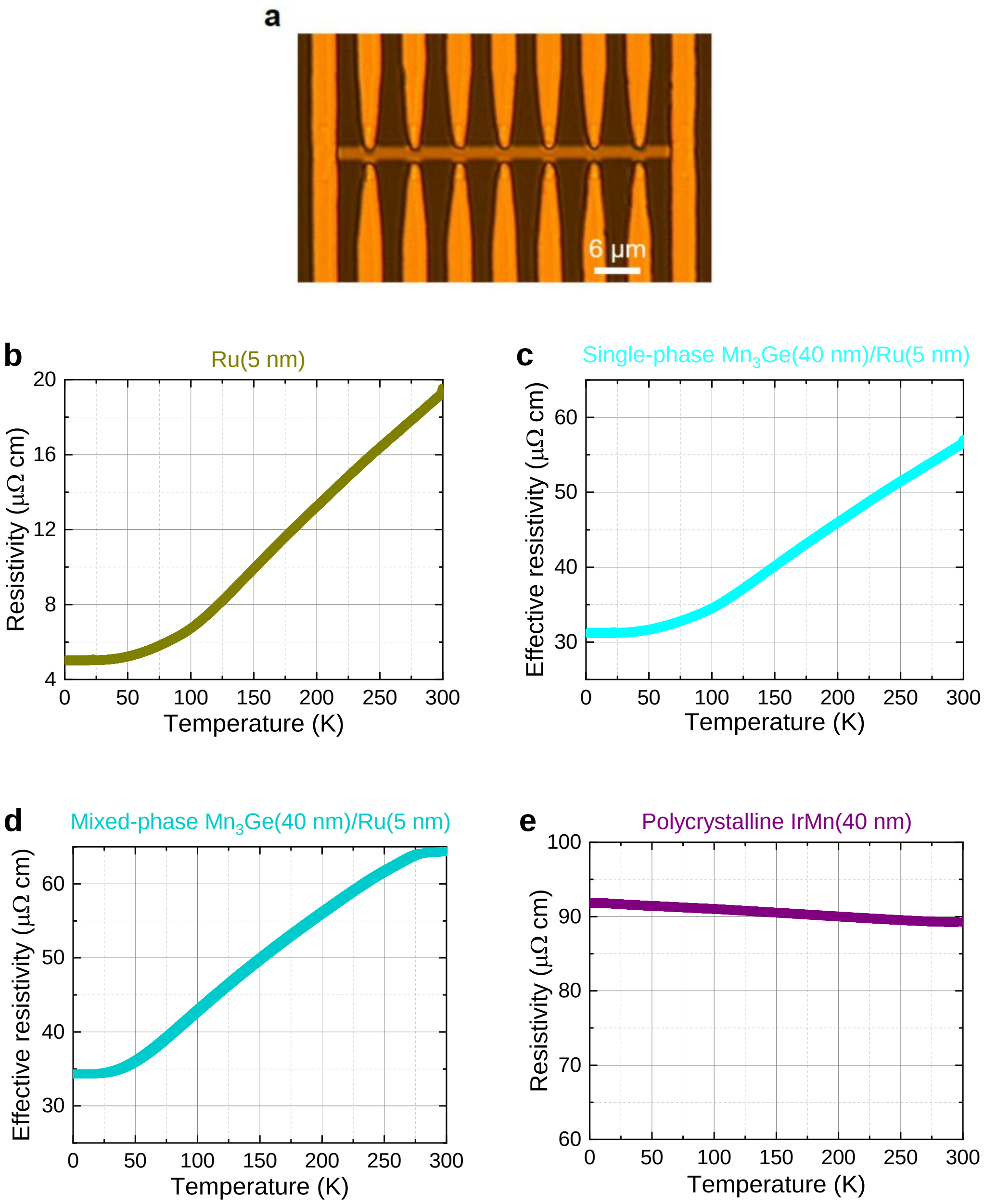

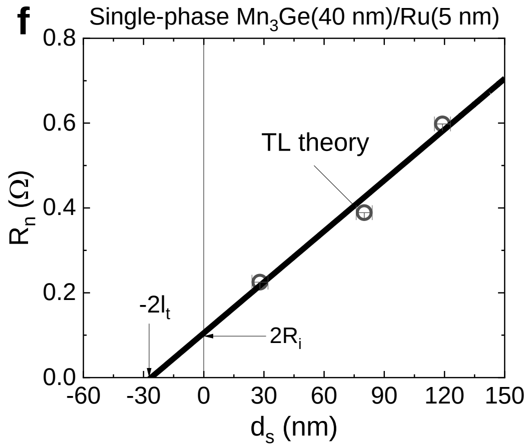

Figure S3. Electrical properties characterized by Hall-bar devices. a, Optical micrograph of the fabricated Hall-bar device. Estimated electrical resistivity versus temperature plots for various thin films of Ru(5 nm) (**b**), single-phase Mn_3Ge(40 nm)/Ru(5 nm) (**c**), mixed-phase Mn_3Ge(40 nm)/Ru(5 nm) (**d**), polycrystalline IrMn(40 nm) (**e**). **f**, Normal-state zero-bias resistance versus d_s plot for the Nb/single-phase Mn_3Ge/Nb JJs (also shown in Fig. 2g, main text). From this plot, one can extract the resistance-area product of Nb/Mn_3Ge interfaces to be 1 mΩ μm^2 and the effective resistivity for the Mn_3Ge(40 nm)/Ru(5 nm) track to be 26 μΩ cm, employing the TL theory[S12].

Section S4. Magnetic field response of an electrically shorting Nb/Mn$_3$Ge/Nb junction.

We present here that if there exists an electrical short-circuit between neighboring Nb electrodes, for instance due to unsuccessful lift-off of the Nb layer (Fig. S4a), how current-voltage *I-V* curves of the electrically shorting Nb/Mn_3Ge/Nb junction change with applying an external magnetic field field $\mu_0 H_\perp$ ($\mu_0 H_\parallel$), perpendicular (parallel) to the interface plane of Nb electrodes. Clearly, the critical current I_c^{short} of the electrically shorting junction barely varies under application of a small (modest) external field $\mu_0 H_\perp$ < 15 mT ($\mu_0 H_\parallel$ < 150 mT) in Fig. S4b (S4c). This contrasts sharply with typical magnetic field interference patterns of Nb/Mn_3Ge/Nb junctions without the short-circuit, which are of significant magnetic field modulation (see Figs. 3 and 4, main text) as would be expected from the true Josephson effect[S14].

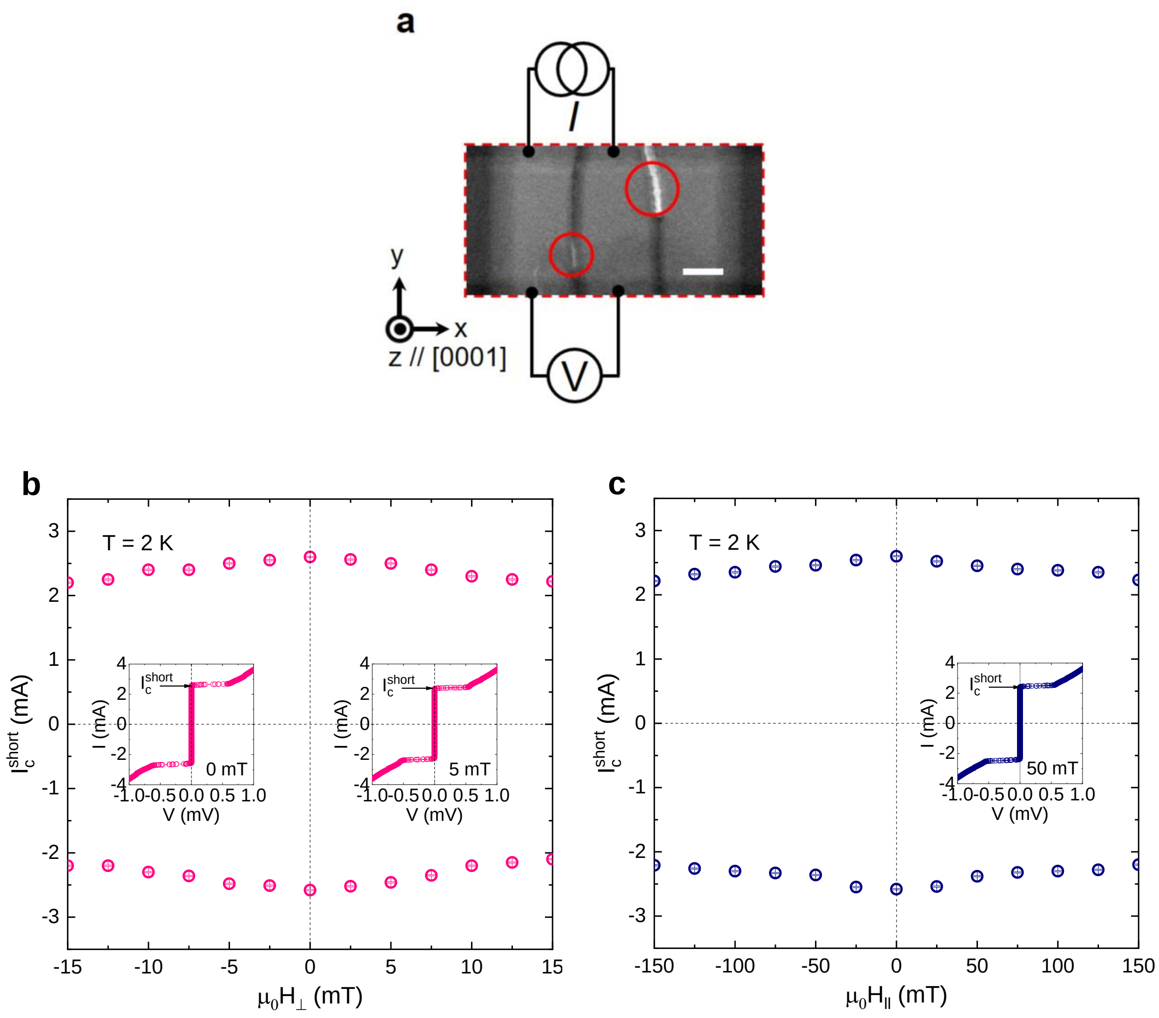

Figure S4. Effect of electrical short-circuits on the magnetic field response. a, Scanning electron micrograph of the Nb/Mn_3Ge/Nb junctions with short-circuits (red circles). The scale bar is 0.5 μm. **b**, Critical current I_c^{short} of the electrically shorting junction as a function of magnetic field $\mu_0 H_\perp$ plot at the fixed T = 2 K. In this measurement, $\mu_0 H_\perp$ is applied perpendicular to the interface plane of Nb electrodes. The left and right insets display current-voltage *I*-*V* curves of the shorted junction at $\mu_0 H_\perp$ = 0 and 5 mT, respectively. **c**, Data equivalent to **b**, but for the magnetic field $\mu_0 H_\parallel$ applied parallel to the interface plane of the Nb electrodes and for the *I*-*V* curve (right inset) taken at $\mu_0 H_\parallel$ = 50 mT.

Section S5. Anomalous Hall effect in the chiral non-collinear AFM Mn_3Ge and the collinear AFM IrMn.

Here we show the crystallographic direction dependence of the anomalous Hall effect for the Mn_3Ge films originating from Berry curvature. For the single-phase Mn_3Ge (0001), we observe

(Fig. S5a) a small but finite anomalous Hall resistivity ρ_{xy} at 300 K when $\mu_0 H$ is applied parallel to the [0001] direction. Although ρ_{xy} for $H \parallel$ [0001] was found to be zero at 300 K in a bulk single-crystalline Mn_3Sn[S15], there were contradicting reports of non-zero ρ_{xy} values (for $H \parallel$ [0001]) for bulk Mn_3Ge[S7,S17]. Very recently, a finite ρ_{xy} has also been observed in a thin film of Mn_3Sn[S18] for $\mu_0 H \parallel$ [0001]. The non-vanishing anomalous Hall response in our single-phase Mn_3Ge (0001) film, which is relatively large in the amplitude compare with bulk Mn_3Ge crystals[S7,S17], might originate from a distortion of the kagome plane as a result of slight atomic shifts/relocations involving a breaking of the ideal $P6_3/mmc$ symmetry of the bulk structure. Interestingly, a sign inversion in ρ_{xy} is observed (Fig. S5a) at 2 K compared to at 300 K, which implies a change of quasi-Fermi level relative to Weyl nodes of the Mn_3Ge semimetal at low temperatures[S19].

In comparison with the single-phase Mn_3Ge (0001), a four times larger ρ_{xy} is observed for the mixed-phase Mn_3Ge $(11\bar{2}0)$ (Fig. S5b) for $\mu_0 H \parallel$ $[11\bar{2}0]$. Qualitatively, a similar orientation-dependent ρ_{xy} has been measured earlier on a bulk single-crystalline Mn_3Ge[S7,S17]. Although one cannot straightforwardly quantify the relative contributions of the $D0_{19}$ hexagonal and the $D0_{22}$ tetragonal phase to the total ρ_{xy} amplitude, it is noteworthy that $\mu_0 H_c$ values extracted from the magnetic curve and AHE measurements do not match each other (~1 T difference at 300 K). This suggests that the epitaxial $D0_{19}$ hexagonal phase dominates the ρ_{xy} amplitude over the polycrystalline $D0_{22}$ tetragonal phase.

The polycrystalline IrMn does not yield any zero-field AHE detectable (Fig. S5c), as expected from a topologically trivial collinear AFM. Note that a linear magnetic field response originates from the ordinary Hall effect.

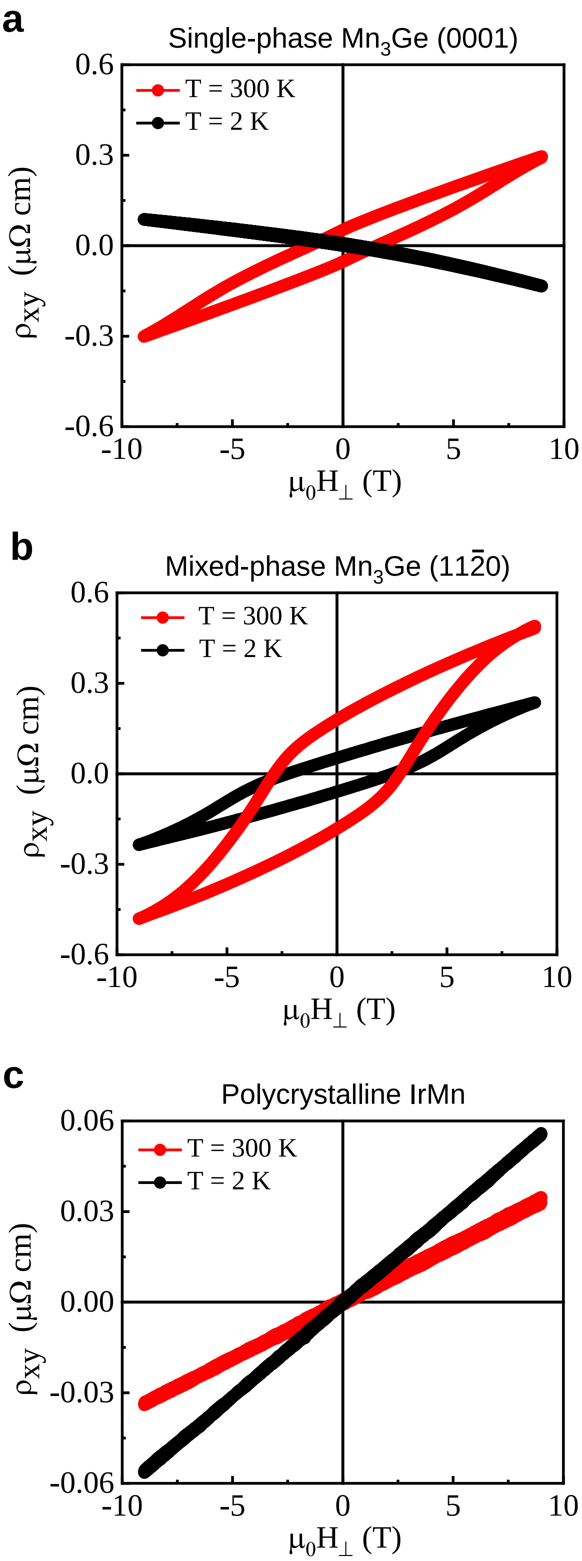

Figure S5. Hall resistivity versus magnetic field *R*-*H* curves of the single-phase $Mn_3Ge(0001)$ (**a**), mixed-phase $Mn_3Ge(11\bar{2}0)$ (**b**), and polycrystalline IrMn (**c**) films at 2 and 300 K. In (**a**) and (**b**), the estimated values should be considered as effective ones for the Hall resistivity as there exists a 5 nm thick Ru buffer layer, to which a certain amount of applied current can shunt.

Section S6. Estimation of the decay length of supercurrents in Nb/IrMn/Nb Josephson junctions.

The interface quality and properties of Josephson junctions (JJs) determine Cooper pair interface transparency and thereby the total amount of proximity-induced Cooper pairs in a non-superconducting spacer. However, we here focus on the fact that that the decay length-scale of supercurrents, which is rather independent of the interface transparency, reflects which type of superconducting correlation (*i.e.* spin-singlet pairs or spin-polarized triplet pairs) is proximity-induced[S20-S22].

We have attempted to estimate the decay length-scale of Josephson supercurrents in Nb/IrMn/Nb junctions by systematically reducing the lateral separation distance d_s of neighbouring Nb electrodes (Fig. S6a). Note that due to technical difficulties in fabricating the *lateral* JJs of $d_s < 20$ nm, we were only able to reduce d_s down to 5–16 nm without electrical shorts by accident (Fig. S6b). For the d_s = 5–16 nm device, small supercurrents ($|I_c|$ = 0.055–0.070 mA) are detectable at zero field for T = 2 K and with an evident magnetic-field modulation. Most importantly, a sudden vanishing of the characteristic voltage $V_c = I_c R_n$ over d_s = 10 nm (Fig. S6l) confirms the *short-range nature* of superconducting proximity effect (*i.e.* spin-singlet pair correlations) in the collinear AFM IrMn spacer, which is fairly consistent with previous reports[S20,S21].

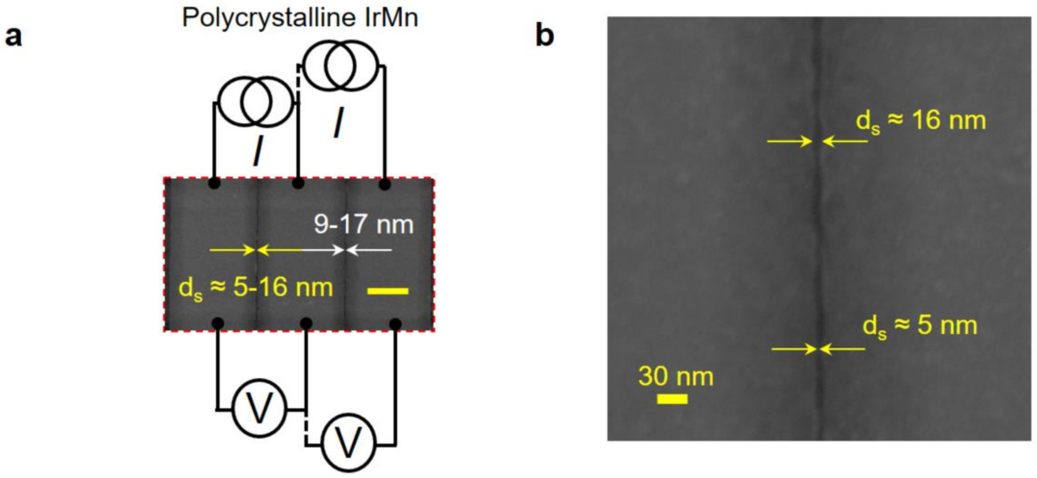

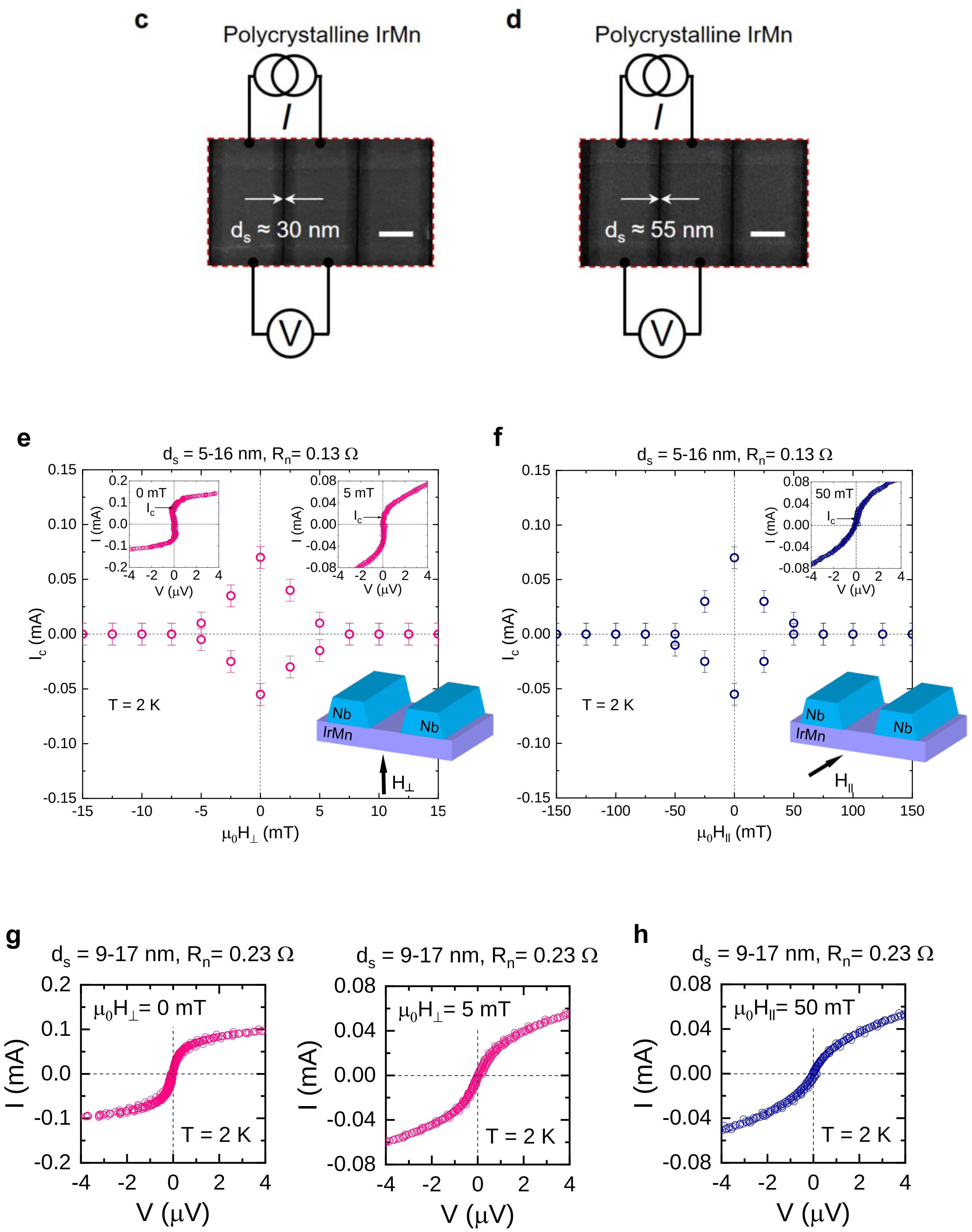

c
Polycrystalline IrMn
I
d_s ≈ 30 nm
V
d
Polycrystalline IrMn
I
d_s ≈ 55 nm
V
e
d_s = 5-16 nm, R_n= 0.13 Ω
0 mT
I_c
I (mA)
V (μV)
5 mT
I_c
I (mA)
V (μV)
I_c (mA)
T = 2 K
Nb
Nb
IrMn
H⊥
μ0H⊥ (mT)
f
d_s = 5-16 nm, R_n= 0.13 Ω
50 mT
I_c
I (mA)
V (μV)
I_c (mA)
T = 2 K
Nb
Nb
IrMn
H∥
μ0H∥ (mT)
g
d_s = 9-17 nm, R_n= 0.23 Ω
μ0H⊥= 0 mT
T = 2 K
I (mA)
V (μV)
d_s = 9-17 nm, R_n= 0.23 Ω
μ0H⊥= 5 mT
T = 2 K
I (mA)
V (μV)
h
d_s = 9-17 nm, R_n= 0.23 Ω
μ0H∥= 50 mT
T = 2 K
I (mA)
V (μV)

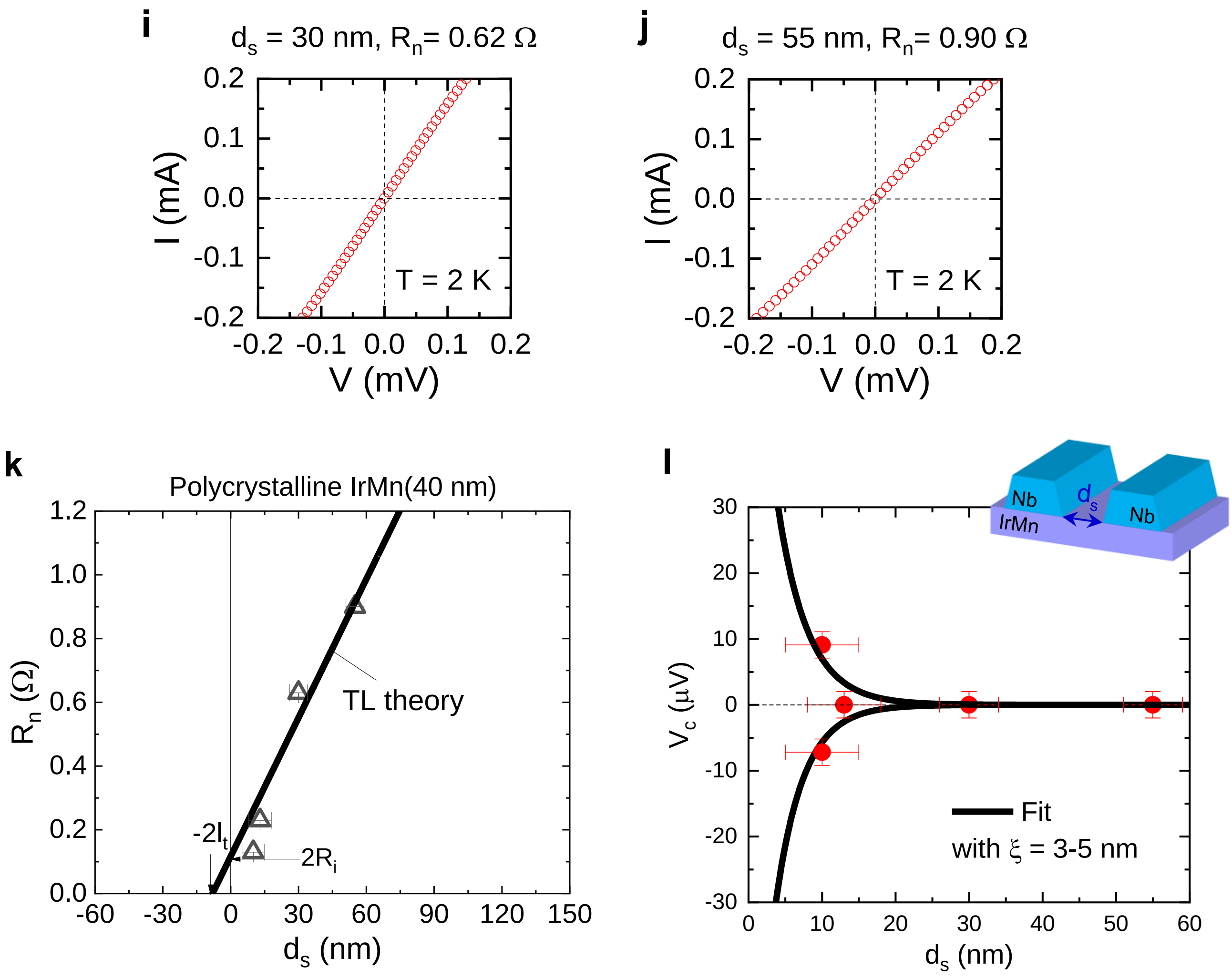

Figure S6. Short-ranged supercurrents through a collinear antiferromagnet. a,**b**,**c**,**d**, Scanning electron micrographs of the fabricated Nb/IrMn/Nb lateral Josephson junction (JJ), where the IrMn spacer is polycrystalline. The scalar bar in **a**,**c**,**d** indicates 0.5 µm. **e**, Magnetic-field interface pattern $I_c(\mu_0 H_\perp)$ when the magnetic field $\mu_0 H_\perp$ is applied perpendicular to the interface plane of Nb electrodes (bottom inset). The top left and right insets display the current-voltage *I*-*V* curves of the d_s = 5–16 nm device, taken around $\mu_0 H_\perp$= 0 and 5 mT, respectively. **f**, Data equivalent to **c** but for the magnetic field $\mu_0 H_\parallel$ applied parallel to the interface plane of the Nb electrodes (bottom inset). **g**,**h**, *I*-*V* curves of the d_s = 9–17 nm device, taken around $\mu_0 H_\perp$= 0 and 5 mT, respectively, and $\mu_0 H_\parallel$ = 50 mT. **i**, Zero-field *I*-*V* curve of the d_s = 30 nm device. **j**, Zero-field *I*-*V* curve of the d_s = 55 nm device. **k**, Normal-state zero-bias resistance R_n of the JJs versus d_s, from which we extract the resistance-area product of Nb/IrMn interfaces to be 0.4 mΩ µm^2 and the resistivity for the IrMn track to be 87 µΩ cm, employing a standard transmission line (TL) theory. **l**, Characteristic voltage $V_c = I_c R_n$ as a function of d_s, from which

the decay length of the Josephson coupling through the IrMn spacer is determined to be less than 10 nm, using an exponential decay function (black curves).

Section S7. Asymmetric hysteretic behaviour in *R*-*H* curves of Nb/Mn_3Ge/Nb JJs.

We have measured the *R versus* $\mu_0 H_\perp$ curves for the d_s = 28 nm device (Fig. 1a, main text). The measured $R(\mu_0 H_\perp)$ curves are basically asymmetric with respect to zero field (Fig. S7a) and their asymmetry ΔR (Fig. S7b) tends to be reversed when we invert the sweep direction of $\mu_0 H_\perp$. This asymmetric hysteretic behavior, which is qualitatively similar to relevant studies [S25,S26], can be more clearly seen in the magnified plot around zero field (Fig. S7c). Even if this result is not conclusive for evidencing spin-polarized triplet pairing states but might be supportive[S25,S26]. Because this makes a link between the magnetic ordering and the superconducting state, this result further supports the spin-polarized triplet pairing interpretation[S25,S26].

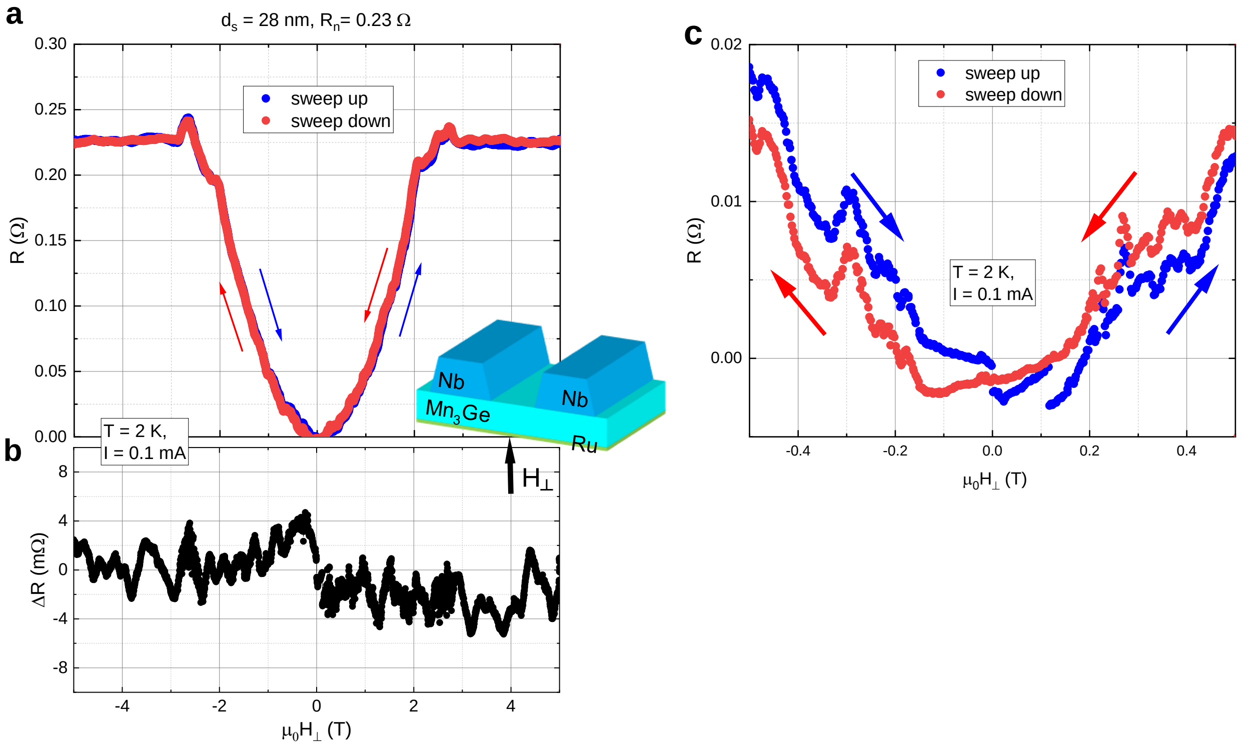

Figure S7. a, Junction resistance *R* versus OOP magnetic field $\mu_0 H_\perp$ plots for the d_s = 28 nm device. **b,** Resistance asymmetry ΔR as a function of $\mu_0 H_\perp$ with $\Delta R = R_{sweep\ up}$ −

$R_{sweep\ down}$. **c**, Magnified plot around zero field.

We also note that asymmetric hysteretic behaviour in *R*-*H* curves of a *ferromagneti*c JJ below T_c reflects, in general, spontaneous magnetization of the FM barrier[S27,S28]. Basically, our *antiferromagnetic* JJs utilize a chiral non-collinear AFM Mn_3Ge whose spontaneous magnetization is tiny (Supplementary Section 2). This accounts for why our chiral AFM JJs reveal a relatively weak asymmetric hysteretic response to external magnetic fields when compared with other relevant studies[S25,S26].

Section S8. Effect of crystal time-reversal symmetry breaking on Josephson coupling.

A very recent theory[S29] has proposed that the crystal time-reversal symmetry breaking in collinear AFMs, arising from collinear antiferromagnetic spin arrangements in combination with *non-magnetic atoms at non-centrosymmetric positions*, can yield a large spontaneous Hall response Here, the key is crystal symmetry breaking and the resultant anisotropic magnetization density.

However, this crystal symmetry breaking mechanism[S29] does not hold for our hexagonal $D0_{19}$-Mn_3Ge thin films whose crystal structure is basically inversion-symmetric (see Fig. 1b,c, main text). In fact, non-collinear AFMs Mn_3X (X = Sn, Ge, Ir, Pt) are the representative material examples for a large anomalous Hall effect via their unique Berry curvature (not via crystal time-reversal symmetry breaking).

The conventional wisdom is that the conversion from spin-singlet Cooper pairs into spin-polarized triplet Cooper pairs via *spin-mixing and spin-rotation processes* requires the existence of *non-collinear magnetism* either at the interface or in the bulk (as in Ref. S21-S22,S30-S33 with FMs). In our case, the straightforward explanation for our findings, supported by the control experiment with the collinear AFM IrMn, is that the unique bulk

properties of the Mn_3Ge (non-collinear AFM spin structure and the resulting fictitious magnetic fields) are at play for the spin-polarized triplet Cooper pair generation. Nonetheless, it would be interesting for future studies to investigate whether the long-range Josephson coupling can also exist in a crystal inversion-asymmetric collinear AFM spacer (*e.g.* RuO_2)[S29] through proper engineering of the non-collinearity.

References

S1. Natl. Bur. Stand. (U. S.), Circ. 539 (1955), IV, 5. Crystal Structure Source: LPF. Am. Mineral. **61**, 177 (1976).

S2. Andrusyak R.I. and Kotur B.Y. "PHASE EQUILIBRIUMS IN THE Sc-Mn-Ge AND Sc-Fe-Ge SYSTEMS AT 870 K". Russ. Metall. (Engl. Transl.) 1991204.

S3. Yamada N., Sakai H., Mori H., Ohoyama T. "MAGNETIC PROPERTIES OF ε-Mn3Ge". Physica B+C (Amsterdam) **149**, 311 (1988).

S4. Mn_3Ge. Status Primary Quality Mark : Prototyping Environment : Ambient Modifications : Reflections Atomic Coordinates : References : 4–5 (2019).

S5. Ogasawara, T., Kim, J. young, Ando, Y. & Hirohata, A. Structural and antiferromagnetic characterization of noncollinear $D0_{19}$-Mn_3Ge polycrystalline film. *J. Magn. Magn. Mater.* **473**, 7–11 (2019).

S6. Markou, A. et al. Noncollinear antiferromagnetic Mn_3Sn films. *Phys. Rev. Mater.* **2**, 051001 (2018).

S7. Nayak, A. K. et al. Large anomalous Hall effect driven by a nonvanishing Berry curvature in the noncolinear antiferromagnet Mn_3Ge. *Sci. Adv.* **2**, e1501870 (2016).

S8. Hong, D. et al. Large anomalous Nernst and inverse spin-Hall effects in epitaxial thin films of kagome semimetal Mn_3Ge. *Phys. Rev. Mater.* **4**, 94201 (2020).

S9. Taylor, J. M. et al. Epitaxial growth, structural characterization, and exchange bias of

noncollinear antiferromagnetic Mn_3Ir thin films. *Phys. Rev. Mater.* **3**, 1–12 (2019).

S10. Kurt, H. et al. Magnetic and electronic properties of $D0_{22}$-Mn_3Ge (001) films. *Appl. Phys. Lett*. **101**, 132410 (2012).

S11. Sugihara, A., Suzuki, K. Z., Miyazaki, T. & Mizukami, S. Magnetic properties of ultrathin tetragonal Heusler $D0_{22}$-Mn_3Ge perpendicular-magnetized films. *J. Appl. Phys.* **117**, 17B511 (2015).

S12. Adel Kalache,1,a Guido Kreiner, Siham Ouardi, Susanne Selle, Christian Patzig, Thomas Höche, and Claudia Felser, Isotropic, high coercive field in melt-spun tetragonal Heusler Mn_3Ge Adel Kalache. *APL MATERIALS* **4**, 086113 (2016).

S13. Schroder, D. K. Semiconductor Material and Device Characterization, 2nd Edition, Wiley-Blackwell (1998).

S14. Barone, A. & Paterno, G. Physics and applications of the Josephson effect. 2nd Edition, John Wiley & Sons (1982).

S15. Nakatsuji, S., Kiyohara, N. & Higo, T. Large anomalous Hall effect in a non-collinear antiferromagnet at room temperature. *Nature* **527**, 212–215 (2015).

S16. Kádárand G. & Krén, E. *Int. J. Mag.* **1**, 143 (1971).

S17. Kiyohara, N., Tomita, T. & Nakatsuji, S. Giant Anomalous Hall Effect in the Chiral Antiferromagnet Mn_3Ge. *Phys. Rev. Appl.* **5**, 064009 (2016).

S18. You, Y. et al. Anomalous Hall Effect–Like Behavior with In-Plane Magnetic Field in Noncollinear Antiferromagnetic Mn_3Sn Films. *Adv. Electron. Mater.* **5**, 1800818 (2019).

S19. Wang, X. et al. Robust anomalous Hall effect and temperature-driven Lifshitz transition in Weyl semimetal Mn_3Ge. *Nanoscale* **13**, 2601–2608 (2021).

S20. Linder, J. & Robinson, J. W. A. Superconducting spintronics. *Nat. Phys.* **11**, 307–315 (2015).

S21. Bergeret, F. S., Volkov, A. F. & Efetov K. B. Long-Range Proximity Effects in

Superconductor-Ferromagnet Structures. *Phys. Rev. Lett.* **86**, 4096–4099 (2001).

S22. Houzet, M. & Buzdin, A. I. Long range triplet Josephson effect through a ferromagnetic trilayer. *Phys. Rev. B* **76**, 060504(R) (2007).

S23. Bell, C., Tarte, E. J., Burnell, G., Leung, C. W., Kang, D.-J. & Blamire, M. G. Proximity and Josephson effects in superconductor/antiferromagnetic Nb/γ−$Fe_{50}Mn_{50}$ heterostructures. *Phys. Rev. B* **68**, 144517 (2003).

S24. Weides, M., Disch, M., Kohlstedt, H. & Bürgler, D. E. Observation of Josephson coupling through an interlayer of antiferromagnetically ordered chromium. *Phys. Rev. B* **80**, 064508 (2009).

S25. Banerjee, N., Robinson, J. W. A. & Blamire, M. G. Reversible control of spin-polarized supercurrents in ferromagnetic Josephson junctions. *Nat. Commun.* **5**, 4771 (2014).

S26. Wang, Y. et al. Proximity-induced spin-triplet superconductivity and edge supercurrent in the topological Kagome metal, K1-xV3Sb5. *arXiv*:2012.05898 (2020).

S27. Chen, C. D.,Yao, Y. D., Lee, S. F. & Shyu, J.H. Magnetoresistance study in Co–Al–Co and Al–Co–Al double tunneling junctions. *J. Appl. Phys.* **91**, 7469–7471 (2002).

S28. Bose, S. K. & Budhania, R. C. Robust coupling of superconducting order parameter in a mesoscale NbN–Fe–NbN epitaxial structure. *Appl. Phys. Lett.* **95**, 042507 (2009).

S29. Šmejkal, L., González-Hernández, R., Jungwirth, T. & Sinova, J. Crystal time-reversal symmetry breaking and spontaneous Hall effect in collinear antiferromagnets. *Sci. Adv.* **6**, eaaz8809 (2020).

S30. Robinson, J. W. A., Witt, J. D. S. & Blamire, M. G. Controlled injection of spin-triplet supercurrents into a strong ferromagnet. *Science* **329**, 59–61 (2010).

S31. Khaire, T. S., Khasawneh, M. A., Pratt, W. P. Jr. & Birge, N. O. Observation of Spin-Triplet Superconductivity in Co-Based Josephson Junctions. *Phys. Rev. Lett.* **104**, 137002 (2010).

S32. Keizer, R. S. et al. A spin triplet supercurrent through the half-metallic ferromagnet CrO2. *Nature* **439**, 825–827 (2006).

S33. Cottet, A. Inducing Odd-Frequency Triplet Superconducting Correlations in a Normal Metal. *Phys. Rev. Lett.* **107**, 177001 (2011).
